# Revealing Missing Human Protein Isoforms Based on *Ab Initio* Prediction, RNA-seq and Proteomics

**DOI:** 10.1038/srep10940

**Published:** 2015-07-09

**Authors:** Zhiqiang Hu, Hamish S. Scott, Guangrong Qin, Guangyong Zheng, Xixia Chu, Lu Xie, David L. Adelson, Bergithe E. Oftedal, Parvathy Venugopal, Milena Babic, Christopher N. Hahn, Bing Zhang, Xiaojing Wang, Nan Li, Chaochun Wei

**Affiliations:** 1 School of Life Sciences and Biotechnology, Shanghai Jiao Tong University, 800 Dongchuan Road, Shanghai 200240, China; 2 Shanghai Center for Bioinformation Technology, 1278 Keyuan Road, Pudong District, Shanghai 201203, China; 3 Department of Genetics and Molecular Pathology, Centre for Cancer Biology, Frome Road, Adelaide, SA 5000 Australia; 4 School of Biological Sciences, University of Adelaide, SA 5005, Australia; 5 School of Medicine, University of Adelaide, North Terrace, Adelaide, SA 5000, Australia; 6 School of Pharmacy and Medical Sciences, Division of Health Sciences, University of South Australia, SA, Australia; 7 ACRF Cancer Genomics Facility, Centre for Cancer Biology, SA Pathology, Frome Road, Adelaide, SA 5000, Australia; 8 CAS-MPG Partner Institute for Computational Biology, Shanghai Institutes for Biological Sciences, Chinese Academy of Sciences, 320 Yueyang Road, Shanghai 200031, China; 9 Department of Clinical Science, University of Bergen, 5021 Bergen, Norway; 10 Department of Biomedical Informatics (DBMI), Vanderbilt University Medical Center (VUMC), 2525 West End Ave, Suite 800, Nashville, TN 37203, USA; 11 Institute of Immunology, Second Military Medical University, 800 Xiangyin Road, Shanghai 200433, China

## Abstract

Biological and biomedical research relies on comprehensive understanding of protein-coding transcripts. However, the total number of human proteins is still unknown due to the prevalence of alternative splicing. In this paper, we detected 31,566 novel transcripts with coding potential by filtering our *ab initio* predictions with 50 RNA-seq datasets from diverse tissues/cell lines. PCR followed by MiSeq sequencing showed that at least 84.1% of these predicted novel splice sites could be validated. In contrast to known transcripts, the expression of these novel transcripts were highly tissue-specific. Based on these novel transcripts, at least 36 novel proteins were detected from shotgun proteomics data of 41 breast samples. We also showed L1 retrotransposons have a more significant impact on the origin of new transcripts/genes than previously thought. Furthermore, we found that alternative splicing is extraordinarily widespread for genes involved in specific biological functions like protein binding, nucleoside binding, neuron projection, membrane organization and cell adhesion. In the end, the total number of human transcripts with protein-coding potential was estimated to be at least 204,950.

Comprehensive gene/transcript annotations are critical reference data for biological studies, especially for genome-wide analyses based on genome annotation. However, alternative splicing (AS) increases the diversity of the transcriptome and proteome tremendously^1^ and makes the task of creating a comprehensive gene/transcript annotation much harder.

AS occurs in organisms from bacteria, archaea to eukarya^2^. Only a few examples can be found in bacteria^3^ and archaea^4^,^5^, but AS is ubiquitous in eukarya^2^. In particular, AS is observed at a higher frequency in vertebrate genomes than in invertebrate, plant and fungal genomes^6^,^7^. In the human genome, the estimated proportion of genes that undergo alternative splicing has been expanded greatly since the start of this century from 38%^8^ to 92%–94%^9^,^10^,^11^. The number of human transcripts generated by AS is estimated to reach 150,000 based on mRNA/ESTs^12^, which is still underestimated based on recent data from the GENCODE project^13^. Other research based on RNA-seq data shows that there are ~100,000 intermediate- to high- abundance AS events in human tissues^9^. The GENCODE Project^13^ aims to annotate all evidence-based gene features including protein-coding genes, noncoding RNA loci and pseudogenes for human. GENCODE V19 contains 196,520 transcripts, of which 81,814 are protein-coding transcripts. However, only 57,005 of them are full length transcripts. Two recent large scale human proteome studies^14^,^15^ expand our understanding of this field. With proteomics data from 17 adult tissues, 7 fetal tissues and 6 purified primary haematopoietic cells, a number of novel proteins were newly identified^14^. In our opinion, a very large proportion of alternative isoforms are still missing, considering the low level of MS/MS spectra of human proteins matching proteins in Refseq^14^. Overall, finding the total number of all transcripts or protein-coding transcripts encoded in the human genome is still an open problem.

RNA-seq is a powerful tool to study transcriptomes and many methods have been developed to reconstruct transcripts from RNA-seq data with^16^,^17^,^18^,^19^ or without^18^,^19^,^20^,^21^,^22^,^23^,^24^ transcript annotations. Some of these methods^16^,^18^,^19^ are based on spliced alignment tools^25^,^26^,^27^,^28^,^29^,^30^. The recent RNA-seq Genome Annotation Assessment Project (RGASP)^31^,^32^ has evaluated 25 protocol variants of 14 independent computational methods for exon identification and transcript reconstruction. Most of these methods are able to identify exons with high success rates, but the assembly of full length transcripts is still a great challenge, especially for the complex human transcriptome^31^. In protein-coding region(CDS) reconstruction methods, the transcript-level sensitivity of CDS reconstruction is no more than 20%^31^, underscoring the difficulty of transcript detection. Direct assembly of transcripts from mRNA-seq reads is not particularly reliable^31^ and these limitations have been reviewed by Martin^33^.

In this paper, we first introduce ALTSCAN (ALTernative splicing SCANner), which was developed to construct a comprehensive protein-coding transcript dataset using genomic sequence only. For each gene locus, it can predict multiple transcripts. We applied it in candidate gene regions in the human genome and 50 RNA-seq datasets from public databases were used to validate the predicted transcripts. Novel validated transcripts are reported and their characteristics are analyzed. In addition, PCR experiments followed by high throughput sequencing were conducted to verify the existence and expression patterns of these novel transcripts. Moreover, the novel transcripts were compared to shotgun proteomics data from 36 breast cancer samples and 5 comparison and reference (CompRef) samples to search for matching novel peptides. We have also evaluated the impact of L1 retrotransposons on the origin of new transcripts/genes. We have used these results to estimate the total number of human transcripts with coding potential.

## Results

### Transcript prediction with ALTSCAN

**Figure 1:**
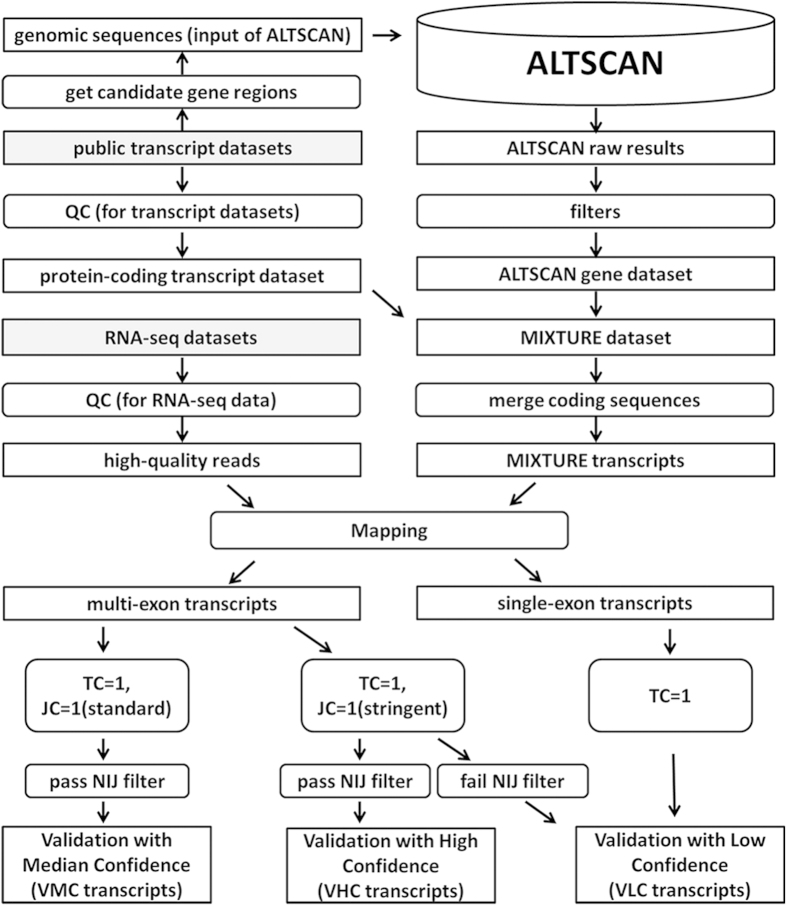
A diagrammatic representation of transcript prediction using ALTSCAN and validation pipeline based on RNA-seq datasets. The upper part shows the pipeline of alternative transcript prediction and the MIXTURE dataset construction. The lower part shows the pipeline for transcript validation with RNA-seq data. The grey blocks indicate raw public data. Candidate gene regions were extracted from various public annotations and then ASs were predicted by ALTSCAN for these regions. Together with the well-annotated KNOWN transcripts, ALTSCAN transcripts were validated with a large number of RNA-seq data. TC is short for transcript coverage and JC is short for junction coverage. The NIJ (novel internal junction) filter was used to check if novel internal junction(s) existed in transcripts (Figure S3). The novel transcript datasets VHC, VMC and VLC were defined as in the figure.

ALTSCAN was developed by extending Viterbi algorithm to predict the most probable N paths (transcripts) for each gene region from the genomic sequence only (see Methods and Figure S1 for details) and applied to human genome sequences (upper part of Fig. 1). As a result, 320,784 transcripts with complete ORFs from 33,945 loci were predicted. Among them, 298,454 transcripts were from 22,606 loci in GENCODE or Refseq gene regions; 8,331 transcripts were from 2,721 loci overlapped with pseudogenes; and almost all remaining transcripts located in repeat-rich regions. Notably, 9,682 transcripts from 7,663 loci overlapped more than 50% (of each transcript) with L1 elements.

**Figure 2:**
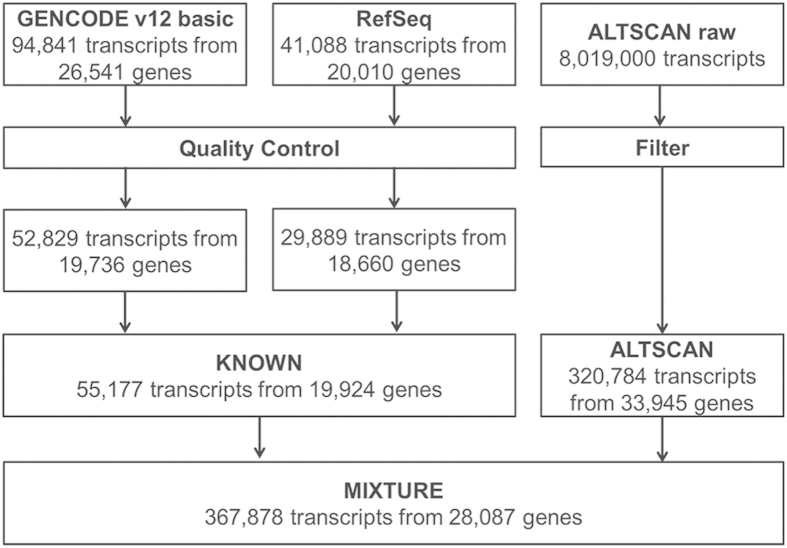
Transcript and gene numbers in dataset construction. The number of transcripts and genes in each dataset is shown. Duplicated GENCODE and Refseq raw transcripts sharing the same coding regions, having internal stop codons or short introns (<20 bp) were removed. Partial-length transcripts were also removed. ALTSCAN raw transcripts sharing the same coding regions were merged and those without complete coding region were removed.

GENCODE and Refseq transcripts were merged to form a dataset named KNOWN (Fig. 2). The KNOWN dataset had an average of 2.76 transcripts per gene while the number from the ALTSCAN dataset was 9.63. 9,780 transcripts from 8,325 genes in the ALTSCAN dataset were consistent with the KNOWN dataset and 84.6% of these consistent transcripts were predicted from sub-optimal paths (Figure S2). Next, the KNOWN and ALTSCAN datasets were then merged to form a dataset called MIXTURE. The MIXTURE dataset contained 367,878 transcripts from 28,087 loci. The reduced gene locus number was due to some relatively long transcripts bridging different clusters of transcripts.

Based on the KNOWN dataset, we compared the performance of ALTSCAN with 3 *ab initio* predictors^20^,^34^,^35^ available in UCSC Genome Browser, as well as 7 predictors^36^,^37^,^38^,^39^ evaluated in RGASP^31^, capable of predicting coding regions (Table 1). As a result, ALTSCAN’s gene-level sensitivity and specificity were 41.8% and 24.4% respectively; much higher than other *ab initio* predictors (the highest one with a sensitivity of 16.8% and a specificity of 14.3%). ALTSCAN’s transcript-level sensitivity and specificity were 17.7% and 3.0% (compared to 6.1% and 14.4% for AUGUSTUS_noRNA, the best *ab initio* predictor in RGASP). This indicated that ALTSCAN could predict many transcripts missed by other *ab initio* predictors. Though the false positive rate of ALTSCAN might be high, we showed that it could be reduced by using RNA-seq data. Integrating RNA-seq data can greatly improve the performance based on the performance of AUGUSTUS with and without RNA-seq data. However, ALTSCAN’s gene- and transcript-level sensitivities are even comparable to the best predictor using RNA-seq data. ALTSCAN’s strategy was to filter the predicted transcripts with diverse RNA-seq data to reduce the false discovery rate, which would be further evaluated with real-time PCR.

In addition, we compared the correct predictions from ALTSCAN, AUGUSTUS_RNA, Exonerate, mGene and Transomics and found 36% (3,522/9,780) of ALTSCAN’s predictions could NOT be detected by the other 4 methods. The numbers for AUGUSTUS_RNA, Exonerate, mGene and Transomics were 13% (1,261/9,105), 21% (1,792/8,453) ,10% (667/6,977) and 8% (569/6,743) respectively. We made similar comparisons among *ab initio* predictors. For those correct predictions, 55% (5,410/9,780) of ALTSCAN, 18% (621/3,369) of AUGUSTUS_noRNA, 15% (401/2,631) of Geneid and 6% (127/2,269) of Genscan transcripts could not be detected by the other 3 methods. Therefore, ALTSCAN could detect many transcripts that other methods missed. It is therefore complementary to current methods.

### RNA-seq validation

**Figure 3:**
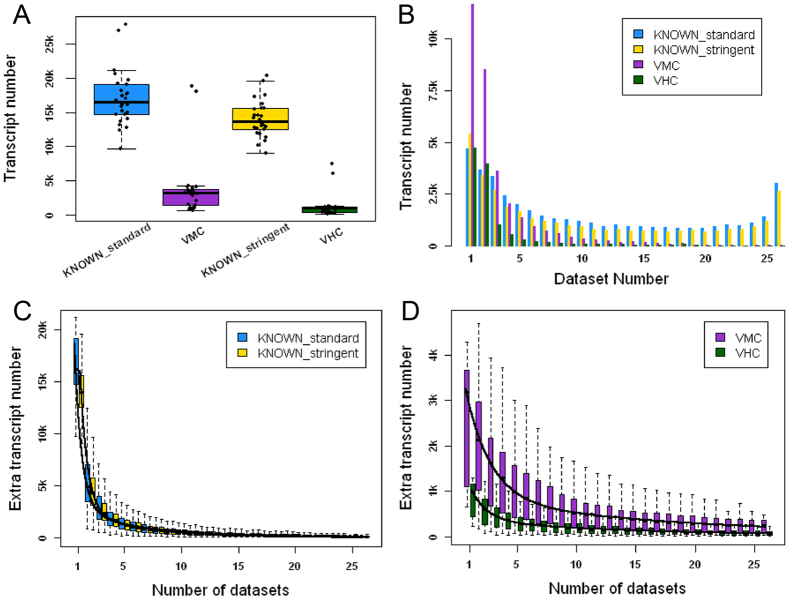
Validation summary of KNOWN and novel transcripts. **A**. shows the number of KNOWN and novel transcripts validated by each RNA-seq dataset using standard or stringent strategy. The highest two points in each group represent validated numbers from GROUP II datasets (RNA-seq data sequenced from the 16 tissues mixture). **B**. shows validated KNOWN and novel transcript numbers using the standard or stringent strategy grouped by numbers of validated datasets. **C** and **D** show the extra numbers of validated KNOWN and novel transcripts using standard or stringent strategy when a new RNA-seq dataset was added. This process was simulated 1,000 times using a bootstrapping strategy.

A pipeline was created to validate the known and predicted transcripts with RNA-seq data (see lower part of Fig. 1). Coding sequences from MIXTURE transcripts were extracted with 100nts upstream of start codons and 100nts downstream of stop codons. These sequence fragments would be validated using high quality RNA-seq reads. We used 26 public datasets (50 RNA-seq runs), which could be grouped into 3 subgroups based on data sources and read lengths (GROUP I, II and III, Table S1), to validate MIXTURE transcripts. We first checked the validation landscape of KNOWN transcripts. Using the standard strategy (see Methods), we could validate about 10 k ~ 20 k multi-exon KNOWN transcripts from each RNA-seq dataset (Fig. 3A and Table S2); and in total, 40,797 multi-exon KNOWN transcripts (73.94% of all KNOWN transcripts, or 76.91% of KNOWN multi-exon transcripts) were validated, of which, 36,128 transcripts were validated from at least 2 different datasets (Fig. 3B and Table S3). Using the stringent strategy, the number of validated transcripts from each dataset were slightly smaller (Fig. 3A and Table S2); in total, 35,037 multi-exon KNOWN transcripts (63.50% of all KNOWN transcripts, or 66.05% of multi-exon KNOWN transcripts) were validated, of which, 29,068 transcripts were validated from at least 2 datasets (Fig. 3B and Table S3). 5,429 (15.50% of 35,037) transcripts were validated from a specific tissue alone, which implied their tissue-specific expression. Furthermore, 1,992 single-exon transcripts (63.70% of single-exon KNOWN transcripts) were also validated.

Next, we checked the validation landscape of novel ALTSCAN transcripts. Using the standard strategy, 31,819 transcripts were validated with medium confidence (the VMC transcripts). 20,124 of these transcripts were validated from at least 2 datasets. Using the stringent strategy, 11,772 transcripts were validated with high confidence (the VHC transcripts). 7,025 VHC transcripts were validated from at least 2 datasets (Fig. 3B and Table S3). 4,747 (40% of 11,772) VHC transcripts were validated from only one dataset. If transcripts validated from less than 5 samples were considered as tissue-specific, we found novel transcripts (VHC or VMC transcripts) had more tissue-specific transcripts than KNOWN (Fisher’s exact test, p-values < 0.001). Figure 3C,D showed the trend of extra numbers of validated KNOWN and novel transcripts when a new RNA-seq dataset was added. In general, more novel transcripts than KNOWN transcripts could be validated when a new RNA-seq dataset was added. For example, more than 70% of the KNOWN transcripts could be validated while less than 30% of the novel transcripts could be validated with 5 randomly selected expression datasets. After the number of datasets exceeded 5, on average, the number of extra transcripts could be validated for the KNOWN_standard transcripts was 344 while it was 726 for the VMC novel transcripts when an RNA-seq dataset was added. Therefore, the expression of novel transcripts tended to be more tissue-specific. In addition, 8,238 transcripts (5,104 single-exon and 3,134 multi-exon transcripts without novel internal junction sites) were also validated as VLC transcripts.

### PCR validation of novel transcripts

**Figure 4:**
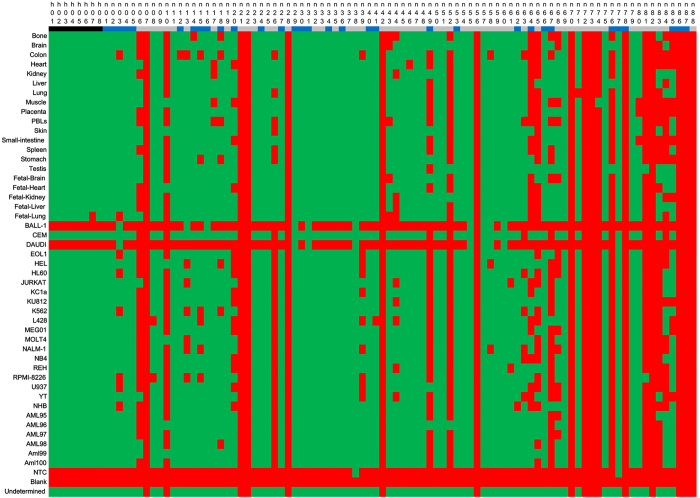
Summary of PCR validation. Black represents house-keeping transcripts; blue represents VHC transcripts; and grey represents transcripts in VMC dataset but not in VHC dataset. Green indicates successful validation, while red represents failed validation. The “blank” line was for a negative control with no RNA used. Reads that could not be classified clearly by the barcodes were merged to “undetermined”.

**Figure 5:**
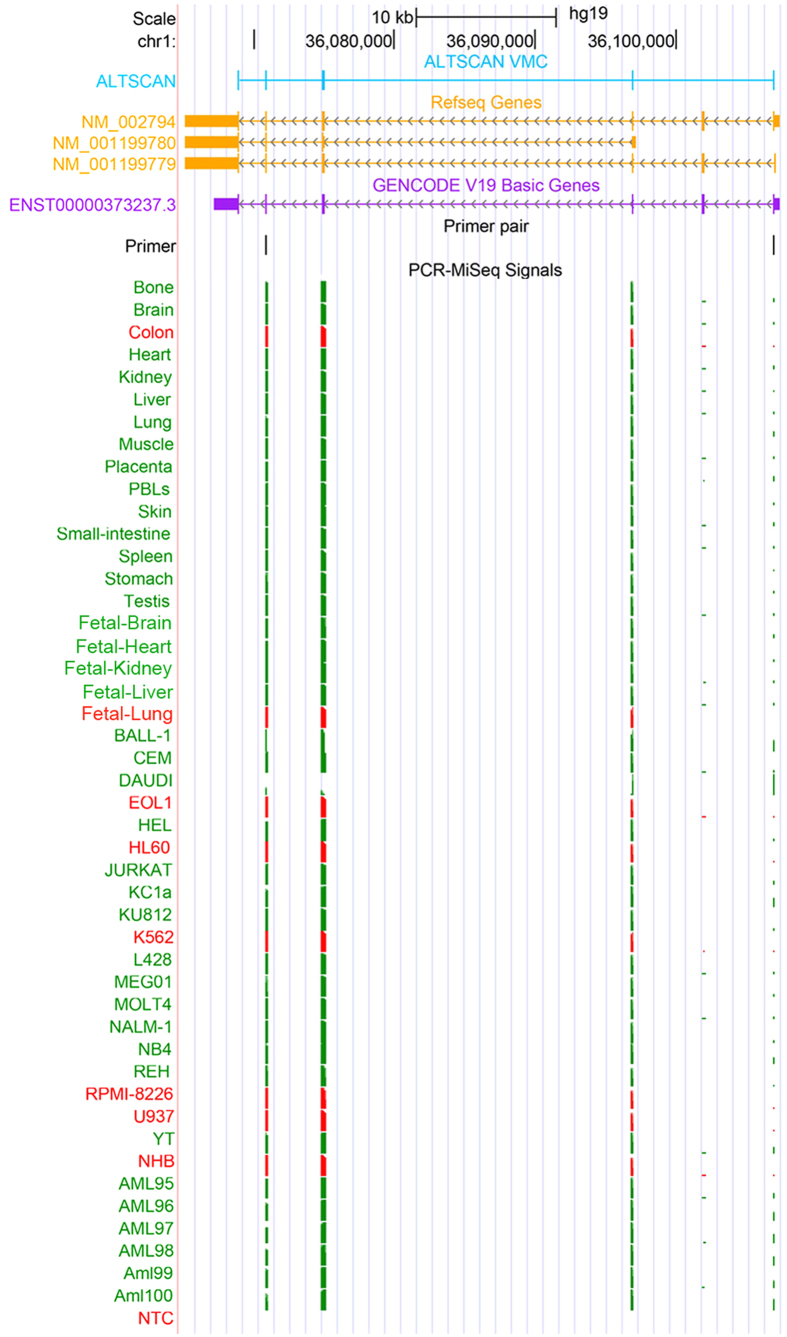
PCR validation of a novel isoform of the PSMB2 gene. The exons of ALTSCAN VMC, Refseq and GENCODE transcripts are shown as boxes in light blue, orange and purple respectively. The second coding exon from the 5’ end was skipped in the ALTSCAN VMC annotation. Primers used for the validation (forward 5’ CTCCAGACATTTCCTAAGGAGTTC3’ and reverse 5’ CAATATTGTCCAGATGAAGGACGGA3’) are shown in black. MiSeq sequencing results of PCR products are shown as PCR-Miseq signals in green and red. Green indicates the transcript was validated in the tissues or cell line and red means the transcript was not validated. This novel isoform of PSMB2 gene was validated in most tissues and cell lines except in colon, EOL1, K562and NHB. In HL60, RPMI-8226 and U937 cell lines, it seemed the novel isoform did exist, but the numbers of reads covering the splicing sites were not large enough to meet our validation criteria. In fetal-lung, NM_001199780 from Refseq annotation seemed to be the only expressed isoform.

We designed primers flanking splice sites of the VMC transcripts, and then randomly selected 88 VMC transcripts for PCR validation (including 32 VHC transcripts) (Table S4). We also designed primers for 8 transcripts of house-keeping genes as positive controls. Real time PCR was used to test these primers on 48 samples (tissues or cell lines, Table S5). Then the products from different samples were mixed and sequenced using the Illumina MiSeq platform. As a result, 8 (8/8 = 100%) house-keeping transcripts were validated by at least one sample, indicating the effectiveness of the PCR validation strategy. For the 88 VMC transcripts, 74 were validated by at least one sample, and the success discovery rate achieved 84.1% (74/88 = 84.1%). For the 32 VHC transcripts, 29 were validated by at least one sample, and the success discovery rate was 90.6% (29/32 = 90.6%). In addition, PCR followed by MiSeq sequencing results showed that the expression of most of these validated novel transcripts was tissue-specific (Fig. 4). For instance, PSMB2 is a gene that influences cooperative proteasome assembly^40^, homologous recombination^41^ and DNA double-strand break repair^41^. Primers were designed to validate the skip of an exon in PSMB2 gene (primer n03 in Fig. 4).This exon skipping event was found in 18 tissues and 20 cell lines and the exon was completely skipped in 7 tissues and 13 cell lines (Fig. 5). The novel isoform was common in different tissues or cell lines but its expression level was lower than the dominating previous known isoform.

### Detection of novel proteins

**Figure 6:**
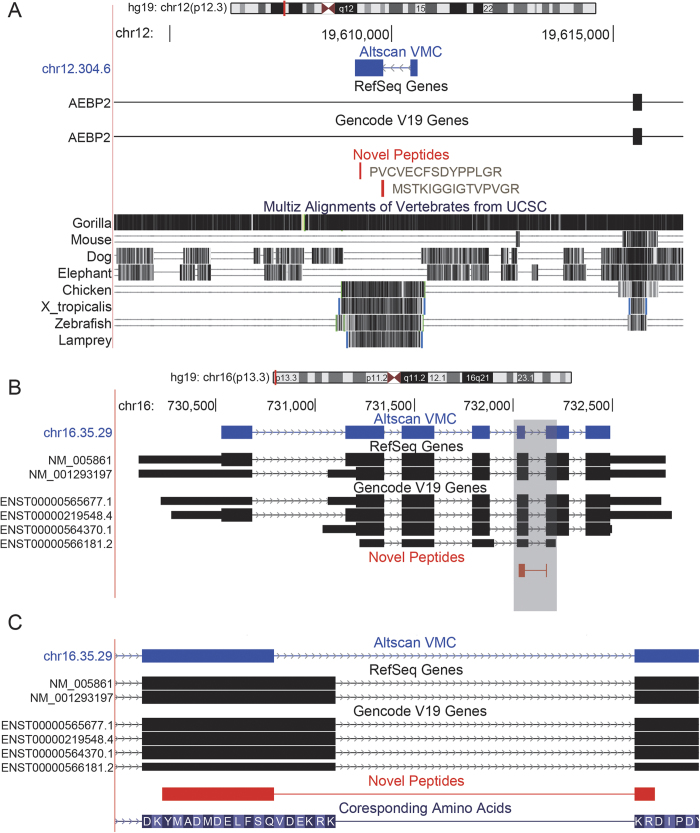
Illustration of novel proteins. **A**. Two novel peptides encoded by a novel gene in the intron of AEBP2 gene. “PVCVECFSDYPPLGR” was detected 4 times and “MSTKIGGIGTVPVGR” once. **B**. Novel peptides detected for STUB1 gene. **C**. Enlarged view of the novel junction (gray area of **B**). The novel peptide “YMADMDELFSQKR” was detected 5 times. Compared to the GENCODE/Refseq protein, 6 amino acids between the tetratricopeptide-like helical domain and the U box domain were removed.

The VHC/VMC transcripts contained complete ORFs and therefore had coding potential. Here we used shotgun proteomics datasets from 36 breast cancer samples and 5 comparison and reference (CompRef) samples to validate the coding potential of these transcripts. The proteomics datasets were used to search a protein database combining Refseq and the VMC transcripts. Candidate novel peptides from VMC transcripts only were further filtered with GENCODE and Swiss-Prot^42^ proteins. As a result, 36 novel proteins supported by at least 2 different peptides including at least 1 novel peptide were detected (Table S8). For instance, we detected two novel peptides encoded in the intron of the AEBP2 gene (Fig. 6A). Moreover, 23 of these 36 novel proteins had at least one novel peptide covering novel splice junction sites. For instance, we detected a novel isoform for the STUB1 gene (Fig. 6B,C). STUB1 protein, a component of E3 ubiquitin ligase, works as a link between the chaperone (heat shock protein 70/90) and proteasome systems^43^. It is also found to be involved in neurodegenerative diseases^44^ and cancers^45^. The novel peptide came from the exon-exon junction of the 5^th^ and 6^th^ coding exons, where alternative donor sites were found. As a consequence, 6 amino acids between the tetratricopeptide-like helical domain and the U box domain were removed from the previously known protein.

### Exploring novel genes

Most of the transcripts in VHC or VMC transcripts were novel isoforms of KNOWN genes. However, 1,053 VMC transcripts from 673 loci (including 485 VHC transcripts from 351 loci) were found outside of KNOWN gene regions (see Methods and Supplementary material). 782 VMC transcripts from 594 loci (including 312 VHC transcripts from 266 loci) remained after the pseudogenes were removed. Almost all the remaining transcripts overlapped with L1 repeat elements. 583 VMC transcripts from 442 loci (including 257 VHC transcripts from 224 loci) were fully covered by single L1 elements (Figure S5A-B). It is known that a small number of human-specific L1 elements are retrotransposition-competent^46^ and undergo AS^47^, and these novel transcripts might be the product of active L1 repeat elements. In addition, 154 VMC transcripts from 128 loci (including 40 VHC transcripts from 32 loci) overlapped partially with L1 elements. 10 out of the 40 VHC transcripts extended out of L1 regions (Figure S5C), indicating their capacity to encroach on other genes. The remaining 30 VHC transcripts bridged two or more repeat elements, including LINEs, SINEs and LTRs (Figure S5D). These repeat elements expanded the complexity of splicing, also known as exonization^48^. Transcripts overlapping partially with L1 elements would appear to be at the very early stage towards the evolution of well-defined functional transcripts and most likely would be dropped in the process of evolution^49^. We had annotated hundreds of such “young” transcripts. The remaining 15 VHC transcripts from 10 loci didn’t overlap with L1 elements (Figure S6). 6 out of the 10 genes shared the same splice sites previously annotated as non-coding RNAs. However, we found complete ORFs in these genes, suggesting their coding potential. Recent human proteome studies have also shown direct evidence that non-coding RNAs can encode peptides^14^,^15^. One of the 10 genes was absent from GENCODE V12 annotation but was added in the V17 version, while the splicing pattern we provided was different. Another one of the 10 genes was conserved in primates and some non-placental vertebrates in its coding region. The remaining two genes were located in the intron or UTR regions of known genes. Similar novel coding regions were also found in recent human proteome studies^14^.

### AS events analysis

**Figure 7:**
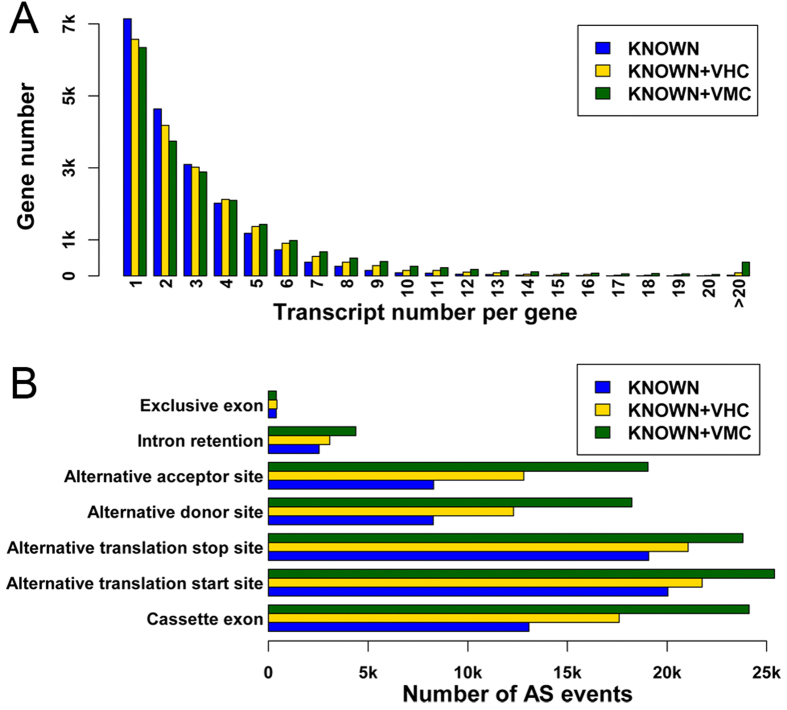
Distribution of alternative splicing in KNOWN and novel datasets. **A**. Distributions of transcript number per gene in KNOWN, KNOWN+VHC and KNOWN+VMC datasets. Genes are grouped by their transcript number. X-axis stands for the group (the number of transcripts per gene), and Y-axis stands for the numbers of genes in each group. All genes with transcript numbers greater than 20 were merged in the same group. **B**. The number of different AS events in KNOWN, KNOWN + VHC and KNOWN+VMC datasets. In order to be comparable, number of AS events involved in each type were measured by using the number of splice sites (see Supplementary material for details).

Recent RNA-seq analysis indicated that 95% of human multi-exon genes are alternatively spliced^11^. However, until now, there were still 5,166 multi-exon genes with only one transcript in the KNOWN dataset. We introduced 31,566 VMC/11,549 VHC transcripts (pseudo-transcript removed), which increased the average transcript number per gene from 2.76 to 4.18/3.30 and decreased the proportion of multi-exon genes with single transcripts from 30.5% to 25.6%/27.2% (Fig. 7A).

We checked the splicing patterns for the validated transcripts. Since our research focused on coding regions, AS events outside of coding regions were ignored. Among all splicing patterns, alternative translation start sites contributed the highest percentage to the complexity of the human proteome followed by cassette exons and alternative translation stop sites as described in KNOWN, KNOWN+VHC and KNOWN+VMC datasets (Fig. 7B and Table S6). However, alternative translation start sites and alternative translation stop sites, similar with alternative promoter and alternative polyA, are known to be mainly induced by transcriptional regulation instead of splicing regulation^50^. Ignoring alternative translation start or stop sites, exon skipping accounted for most novel AS, which is consistent with our knowledge^11^,^50^. Compared with KNOWN transcripts, we found that exon skipping, alternative donor sites and alternative acceptor sites accounted for more proportion of AS in KNOWN+VMC or KNOWN+VHC transcripts (p-values of Fisher’s exact test < 0.001). Alternative splice acceptor or donor sites are known to be an intermediate state between constitutive and alternative cassette exons and therefore might be prevalent in the human proteome^7^.

### Functional analysis

GO (Gene Ontology) enrichment analysis is widely used in biological studies and the background distribution of GO functions is critical in analysis procedures. We carried out GO annotation for these novel transcripts. We found that the function distribution of the VHC/VMC transcripts was quite consistent with that of the KNOWN transcripts (Figure S8A-F). The Pearson correlation coefficients of function distributions between VHC and KNOWN transcripts were 0.985, 0.950 and 0.967 in biological process, molecular function and cellular component level respectively (Figure S8G-I). The corresponding coefficients between VMC and KNOWN transcripts were 0.989, 0.970 and 0.988, respectively (Figure S8J-K). These results indicated that novel transcripts predicted by our methods had similar functional distributions compared with known transcripts.

In order to investigate the influence of AS on specific biological process, we examined whether transcript numbers of genes were related to biological functions and pathways. Enrichment analysis of genes with a high number (>5) of transcripts showed AS was enriched in specific molecular functions (binding, especially protein binding and nucleoside binding and enzyme regulatory activities like transcription cofactor activity), cellular components (neuron, membrane-related locations and cell junction) and biological process (regulation of small GTPase mediated signal transduction, vesicle-mediated transport and membrane invagination) (Table S7). However, no enriched KEGG pathways were found. This might be because although one gene with different biological functions had AS bias, the overall AS bias for all genes involved in the pathways was not significant.

### Estimation of the total number of transcripts with coding potential

**Figure 8:**
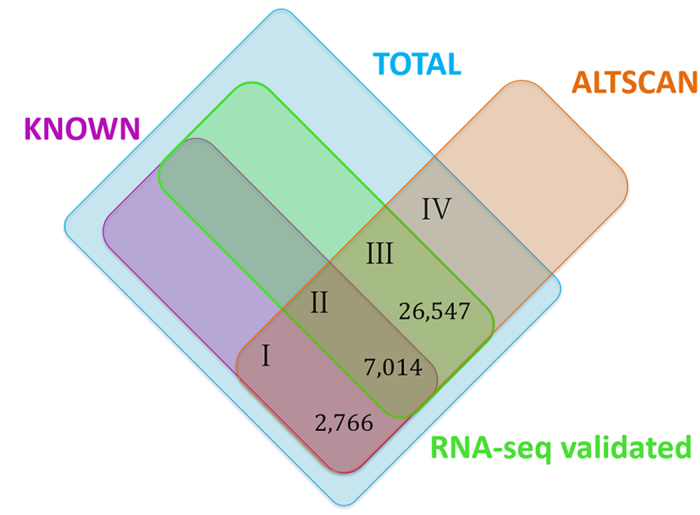
Estimation of the number of transcripts with coding potential. Datasets are illustrated in different colors. “TOTAL” means the total transcript dataset whose transcript number was to be estimated. Different datasets were represented by different numbers. *I* represents transcripts in KNOWN and ALTSCAN datasets but not been validated by RNA-seq data used in this study; *II* represents transcripts in KNOWN and ALTSCAN datasets and validated by RNA-seq data used in this study; *III* represents VHC or VHC+VMC transcripts; *IV* represents novel but real transcripts in ALTSCAN datasets that have not been validated by RNA-seq data used in this study.

In spite of the advancement of RNA-seq technology, estimating the total number of human protein-coding transcripts is still an open problem. Many transcripts are expressed at low levels or in a temporally and spatially specific way. As a consequence, they are difficult to discover making it difficult to estimate the total number of human proteins. ALTSCAN can be used as an *ab initio* predictor and its sensitivity is no influenced by expression levels. Therefore, we may assume that ALTSCAN’s calculated sensitivity based on known transcripts is equal to the value calculated by considering the undiscovered transcripts (see Fig. 8 and Methods for details). Based on this assumption, we estimated the number of human transcripts with coding potential to be at least 204,950.

## Discussion

AS expands the functional repertoire of the human genome, but only a small proportion of AS has been experimentally characterized. A comprehensive gene annotation is critical for genome-wide analysis, cis-regulatory element finding, hereditary disease studies and nearly all biological science studies. Detecting all genes and transcripts for human and other model organisms is one of the long term goals of biological research. In this paper, we have introduced ALTSCAN, and demonstrated that by directly predicting many transcripts in a single locus from the genomic sequence and filtering the predictions with RNA-seq data, we were able to generate a large number of novel protein-coding transcripts.

ALTSCAN’s transcript-level sensitivity is 17.7% while the corresponding number is 6.1% for the best existing *ab initio* predictor. It demonstrates that ALTSCAN’s multi-layer Viterbi algorithm is able to detect more transcripts. Recently, RGASP assessed many transcript reconstruction methods^31^ and the predictions from different methods have been evaluated with expressed transcripts from GENCODE v3c only, instead of all known transcripts in public databases. In our evaluation, the KNOWN dataset (GENCODE and Refseq dataset) was used as the annotation dataset. In RGASP, the best transcript level sensitivity for CDS reconstruction was 19.8% (16.5% in our evaluation results, due to the increased number of annotated transcripts by merging the Refseq and GENCODE datasets). By comparing the correct predictions from different programs, we have found that ALTSCAN can detect many transcripts that other methods may miss. Therefore, ALTSCAN is complementary to existing methods. Recently, single molecule real-time (SMRT) sequencing was used to obtain transcriptome data from 20 human organs and tissues^51^,^52^. From these data, 11,833 transcripts not included in GENCODE were created (from authors Tilgner H. and Snyder MP.^51^,^52^). 11,084 of them were labeled as “protein-coding”. We compared the VMC transcripts with these 11,084 novel protein-coding transcripts. As a result, 2,214 VMC transcripts were supported, which meant all the splice junctions of a VMC transcript were consistent with a SMRT transcript. The sensitivity on this SMRT novel transcript data was about 20% (2,214/11,084), which was similar to ALTSCAN’s performance in the KNOWN dataset. The conservation level of transcripts in VMC, Refseq, GENCODE and novel SMRT transcripts were similar when they were compared to the mouse genome (mm10). This indicated that our assumptions used to estimate the overall number of human transcripts were reasonable.

Despite its excellent ability to detect novel transcripts with high confidence when integrated with RNA-seq data, ALTSCAN had some limitations. First, our results from ALTSCAN were still far from exhaustive due to the limitations of the algorithm and computing capability. In our extended Viterbi algorithm, the average transcript number discovered showed no sign of decreasing even at a depth of 250, which suggested that this depth was still insufficient to detect all transcripts. In addition, the initial ALTSCAN prediction before the RNA-seq filter contained many redundant transcripts.

In addition, our RNA-seq studies focused on validation of candidate transcripts without exploring the whole expression profiles from different tissues. Relatively strict criteria were used to remove the mapping errors of RNA-seq reads to the reference genome. Since only limited number of tissues/cell-lines were used in this study, more transcripts might be validated if more tissues or cell-lines were added to PCR validation. Therefore, the false discovery rate of our pipeline was at most 9.4% VHC transcripts) or 15.9% (VMC transcripts).

Recent data from the ENCODE project indicated that about three-quarters of the human genome was capable of being transcribed^53^, which increased the importance of mapping of splice junction reads when validating spliced gene structures. Therefore, we paid more attention to the validation of junction sites instead of “transcription”. In order to get reliable prediction results, the sequencing depth and different parameters in our validation pipeline were assessed for their impact on the number of validated transcripts. Results showed that shorter reads required more strict validation parameters, and deeper sequencing depth could help validate more novel transcripts.

We also found that hundreds of transcribed L1 elements may be still active. L1 elements provided many potential splice sites^54^. After their insertion in new locations of the genome, they could alter the coding potential of nearby nucleotides with their active splice sites. Although this might “break” a nearby gene, it can be a tremendous source of exonization and a driving power of evolution. In addition, we detected several novel proteins encoded by L1 elements in both cancer and CompRef samples (Table S8).

The identification of all human proteins is an important and unsolved question. Our novel transcripts can help detect novel proteins. Mass spectrometry (MS) and ribosome profiling (RP)^55^ methods can be used to study the proteome. MS detected peptide segments from a candidate protein pool; and RP provided only short portions of RNAs that were bound to ribosomes. Recent human proteome studies took a big step towards annotation of all human proteins, however, it was far from complete, mostly due to isoforms derived from AS, which often differed by only several peptides near the corresponding splice sites. Therefore, novel proteins were very difficult to discover by both methods^49^. We have detected 62 novel proteins missing in Refseq. 29 of these 62 proteins have novel peptides covering splice junctions. Overall, 9 of the 62 proteins have been annotated in both GENCODE and Swiss-Prot. Among the 62 proteins, 5 and 11 of them have been annotated in GENCODE only and Swiss-Prot only respectively. Therefore, the final number of novel proteins is 61 – 9 – 5 – 11 = 36. To our view, finding 36 novel proteins in one tissue (41 samples) is quite effective. Surprisingly, 24 of the novel proteins have novel peptides covering novel splice junctions, indicating the capability of our method to detect novel transcripts especially for those with novel splice sites. In short, our work is an effective supplement to existing methods and will help to build a more comprehensive human protein-coding gene annotation.

In conclusion, we have developed a novel system to predict protein-coding transcripts by integrating *ab initio* prediction and filtering with RNA-seq data; and we have detected and validated 11,549 ~ 31,566 transcripts with complete ORFs at a FDR of 9.38% ~ 15.9%. In contrast to known transcripts, these novel transcripts are highly tissue-specific. We estimate the total number of full length transcripts to be no less than 200 thousand, indicating that majority of the protein-coding transcripts are still missing in the current databases. In addition, 36 novel proteins were detected. Furthermore, we found that L1 elements have a far greater impact on the origin of new transcripts/genes than previously thought. Alternative splicing is extraordinarily widespread for genes involved in some basic biological functions.

## Materials and Methods

Detailed methods can be found in Supplementary material. Here we described materials and methods briefly.

### ALTSCAN

ALTSCAN utilized an extended Viterbi algorithm. The top N value(s) were kept in each step so that the top N path(s) would be generated, which enabled the scanner to predict multiple transcripts for one gene. N was set to 250 for most ALTSCAN inputs. Figure S1 shows how the extended Viterbi algorithm worked.

### ALTSCAN prediction for the human genome

In practice, candidate gene regions were extracted from the human genome as the input to ALTSCAN (upper part of Fig. 1). The candidate gene region included the regions of known genes, SIB genes, and NSCAN predicted genes. The known genes included GENCODE basic V12 genes, which were derived from the HAVANA manual annotation process and Ensembl automatic annotation pipeline and Refseq genes^56^. SIB genes^57^ were genes with supporting evidence of at least one GenBank full length RNA sequence, one Refseq RNA, or one spliced EST. SIB genes were used to create regions with mRNA or EST evidence. In addition, NSCAN predicted genes were those predicted genes from multiple-genomes. GTF files were collected for all these gene datasets, and a total of 33,480 sequences including a padding length of 5,000 bps both downstream and upstream of genes were extracted from the human genome (hg19). ALTSCAN was run on these regions and raw results were filtered and clustered to ensure each transcript had a unique coding sequence. Finally, 320,784 transcripts with unique complete coding regions from 33,945 genes made up the ALTSCAN prediction for the human genome. Details of ALTSCAN’s prediction for the human genome are described in Supplementary material.

### Assessment of coding region (CDS) prediction

We evaluated the performance of tools for CDS prediction including 4 *ab initio* predictors (ALTSCAN, Genscan^35^, Geneid^34^ and AUGUSTUS^20^) and 7 predictors using RNA-seq data (AUGUSTUS^37^, Exonerate^38^, mGene^36^, mTim, NextGeneid, Transomics and Tromer^39^) based on the KNOWN annotation. Predictions from AUGUSTUS_no_RNA and all predictors using RNA-seq data were downloaded from RGASP^31^,^32^. The evaluation on gene-, transcript- and exon-level was achieved with the tool RGASP.jar provided by RGASP.

### RNA-seq validation

We collected 50 RNA-seq runs from the Illumina Human BodyMap2 project and ENCODE project. Different runs of a biological sample were merged to 26 datasets. These datasets were further classified into to 3 groups based on data source and sequencing features (see Table S1). We created a pipeline (lower part of Fig. 1) to validate known and predicted transcripts with these RNA-seq data. Quality control of RNA-seq data was carried out using the NGSQC^58^. Coding sequences from MIXTURE transcripts were extracted with 100nts upstream of start codons and 100nts downstream of stop codons. These coding fragments formed the mature transcript dataset. High quality reads were mapped to the mature transcript dataset using Bowtie^59^. A splicing junction site was covered if and only if at least *M* read(s) covered both sides of the adjacent exons with no less than *L* nts on each side. We used two strategies in our splice junction site validation: the standard strategy (*L* = 10 and *M* = 1) and the stringent strategy (*M* > 5 and *L* > 7, Figure S4). In addition, novel validated transcripts (in ALTSCAN but not in KNOWN dataset) were further filtered by the NIJ (novel internal splice junction, Figure S7) filter and grouped into VHC (validation with high confidence), VMC (validation with median confidence) and VLC (validation with low confidence) datasets (Fig. 1).

### PCR validation of novel transcripts

Primers were designed with Primer3^60^. Real-time PCR was conducted using EvaGreen on the Biomark System (Fluidigm). PCR products from the same samples were mixed and barcodes were added. Finally, samples were pooled and sequenced using an Illumina MiSeq sequencer (see PCR experiment part in Supplementary material).

### Detection of novel proteins

Shotgun proteomics data of 36 breast cancer samples (900 raw files) and 5 CompRef samples (125 raw files) generated by Clinical Proteomic Tumor Analysis Consortium (NCI/NIH) were used in this study^61^. The 5 CompRef samples were used to monitor the consistency of laboratory protocols and mass spectrometry instrument performance. The mass spectrometry raw data were compared against a combined database including Refseq protein sequences, VMC protein sequences and a decoy database with all protein sequences reversed, using the X!Tandem search engine^62^. The false discovery rate (FDR) was set at 10^−6^ as previously described^63^. Peptides that could be scored according to the VMC transcripts but could not be scored according to the Refseq transcripts were identified as the preliminary novel peptides. Proteins that could be mapped by at least two identified unique peptides including at least one novel peptide were defined as candidate novel proteins. These preliminary peptides were further aligned to GENCODE (version 12) and Swiss-Prot^42^ (downloaded on Dec. 1, 2014) proteins to get the final novel peptides using NCBI BLAST (blastp).

### AS event analysis

AS events were classified into seven categories and were detected with methods described in Supplementary material and Figure S7.

### Functional analysis

Enrichment analysis was carried out with DAVID^64^ and iGepros website^65^. Enrichment p-values were adjusted using the Benjamini-Hochberg method.

### Estimation of the total number of transcripts with coding potential in human

In order to estimate the total number of transcripts with coding potential in human, we assumed the sensitivity of ALTSCAN evaluated using known transcripts was equal to the sensitivity for undiscovered transcripts. The relationship between the datasets (I, II, III and IV) is shown in Fig. 8. ALTSCAN’s sensitivity based on known transcripts was calculated as in equation (1).


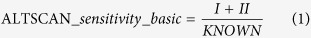


ALTSCAN’s sensitivity including previously undiscovered transcripts can be described as in equation (2).


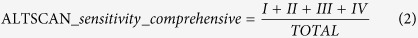


As a result, the total number of transcripts with coding potential in human can be described as


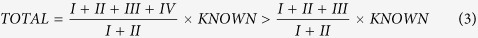


where *I* + *II* = 9780 and *III* = 31,566 × 84.1% = 26,547 (VMC transcript number multiplied by accuracy estimated from PCR validation). *IV* represents novel transcripts predicted by ALTSCAN without RNA-seq validation. We found that using GROUP II data only (sequenced from mixtures of 16 tissues), 30,433 VMC transcripts could be obtained. The other 24 datasets contributed additional 1,133 transcripts; indicating that *IV* would be a small proportion of the total transcripts.

## Additional Information

**How to cite this article**: Hu, Z. *et al.* Revealing Missing Human Protein Isoforms Based on *Ab Initio* Prediction, RNA-seq and Proteomics. *Sci. Rep.* **5**, 10940; doi: 10.1038/srep10940 (2015).

## Supplementary Material

Supplementary Information

## Figures and Tables

**Table 1:** Assessment of protein coding region prediction based on the KNOWN dataset.

Predictor type	Predictor	Gene		Transcripts		Multiple transcripts per gene
		sensitivity	specificity	sensitivity	specificity	
*ab initio*	ALTSCAN	41.8%	24.4%	17.7%	3.0%	yes
	Genscan^b^	11.3%	2.2%	4.1%	2.2%	no
	Geneid^b^	13.0%	8.0%	4.7%	8.1%	no
	AUGUSTUS_noRNA^a^	16.8%	14.3%	6.1%	14.4%	yes
*RNA-seq*	AUGUSTUS_RNA^a^	41.6%	49.8%	16.5%	44.3%	yes
	Exonerate^a^	36.1%	23.3%	15.3%	27.3%	no
	mGene^a^	34.6%	11.2%	12.6%	11.3%	yes
	mGene_graph^a^	31.5%	47.6%	12.6%	29.4%	yes
	mTim^a^	17.3%	53.4%	6.6%	43.0%	yes
	NextGeneidAS^a^	24.8%	28.8%	9.6%	30.9%	no
	NextGeneidAS ab-initial^a^	24.8%	26.8%	9.6%	28.8%	no
	Transomics^a^	33.4%	19.9%	12.2%	20.2%	no
	Tromer^a^	3.5%	1.5%	1.3%	0.6%	yes

^a^Predictions of these methods were derived from RGASP directly.

^b^Predictions of these methods were downloaded from UCSC Genome Browser.
