# Supplementary material for: Revealing Missing Human Protein Isoforms Based on Ab Initio Prediction, RNA-seq and Proteomics
Source: Sci Rep. 2015 Jul 9;5:10940. doi: 10.1038/srep10940 (PMC4496727; doi:10.1038/srep10940)
Supplement: Supplementary Information [file srep10940-s1.pdf]

Supplemental Materials

Revealing Missing Human Protein Isoforms Based on *Ab Initio* Prediction, RNA-seq and Proteomics

Zhiqiang Hu, Hamish S. Scott, Guangrong Qin, Guangyong Zheng, Xixia Chu, Lu Xie, David L. Adelson, Bergithe E Oftedal, Parvathy Venugopal, Milena Babic, Christopher N Hahn, Bing Zhang, Xiaojing Wang, Nan Li, Chaochun Wei

CONTENTS

1 Introduction 1
2 ALTSCAN 1
2.1 Algorithm 3
2.2 Training and prediction 7
2.3 Evaluation 8
2.4 Deficiency 10
3 Dataset construction 10
4 RNA-seq validation 11
4.1 RNA-seq data information 11
4.2 Validation strategies 14
4.3 Parameter selection 15
4.4 Validation landscape 18
5 PCR validation 20
5.2 Real-time PCR experiment 22
5.3 Validation from sequencing results 25
6 Exploring novel genes 26
7 AS analysis 30
7.1 Detection of AS events 31
8 GO/KEGG analysis 33
8.1 GO analysis 33
8.2 Enrichment analysis 35
9 Detection of novel proteins 37
9.1 Annotated dataset construction 37
9.2 Database search 37
9.3 Novel peptides and novel proteins identification 38
11 Accessing software and data 39
12 References 39

1 Introduction

In this Supplementary material we give technical details regarding the detection, validation and characterization of novel human transcripts. First, we introduce ALTSCAN system in detail. Next, we give additional information for validation and consequent analysis described in the manuscript. Finally, we showed how the data mentioned in this paper would be accessed.

2 ALTSCAN

ALTSCAN (ALTernative splicing SCANner) is developed to find all possible exon-intron structure for coding genes. ALTSCAN incorporates transcriptional, translational, and splicing signals, together with length distributions of exons, introns and intergenic regions to predict multiple transcripts for each gene. When calculating the probability of a transcript, an extended Viterbi algorithm was utilized. Here we first presented details of ALTSCAN algorithms (Figure S1), and then showed the processes of training and prediction.

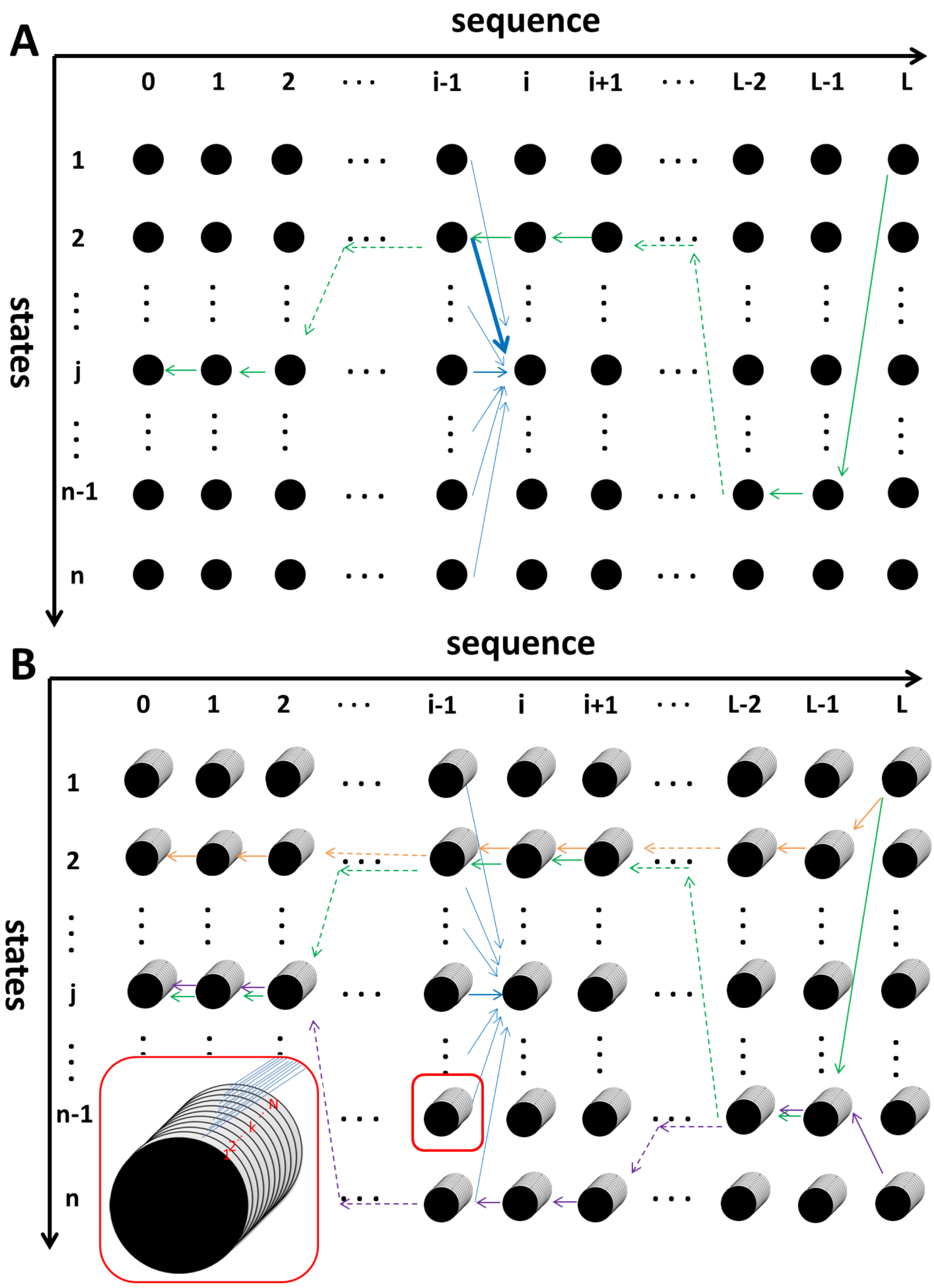

Figure S1. ALTSCAN algorithm. ALTSCAN utilizes an extended Viterbi algorithm. Viterbi algorithm, which is a variation of dynamic programming, finds the most probable path in HMM/GHMM. A "trellis" is used to keep all the values in dynamic programming. **A** shows the "trellis" used in a traditional Viterbi algorithm. **B** shows the "trellis" used in ALTSCAN. A cell keeps an array of values instead of a single value, as shown in the enlarged view. The maximum top *N* values will be calculated from arrays of values and top N pointers will be kept. Finally, the most probable *N* paths will be reconstructed by tracing back.

2.1 Algorithm

Generalized Hidden Markov Model (GHMM, also called Hidden Semi-Markov Model) has been utilized in gene structure prediction since 1997[1]. Compared with Hidden Markov Model (HMM), whose states follow geometric length distribution, GHMM supports general length distributions for its states. This is necessary for human gene structure prediction because of the non-geometric length distribution of its exons. On the other hand, the GHMM involves a more complex algorithm, requiring a recursion which searches back at each position over all possible previous positions. If the length of the sequence is L and the state number is N, then the computing complexity will be $O(N^2L^2)$. Obviously, the algorithm must be simplified due to the large size of L to make this approach practical. Burge proposed a simplified method[1] based on several assumptions: 1) States are grouped into 2 classes, coding states and non-coding states; 2) Coding states have durations less than D, and will be still treated using general length distribution; 3) Non-coding states follows geometric length distributions and sequence generating models which are “factorable”, for instance, the emission probability E for type i to generate sequence s can be described as $\mathrm{E}_i(s_{a,c}) = \mathrm{E}_i(s_{a,b})\mathrm{E}_i(s_{b,c})$, where a, b and c are sequence positions and $s_{x,y}$ represents the sequence segment of sequence s from position x to y.

For HMM/GHMM model, the optimization problem is efficiently solved by the Viterbi algorithm. We first describe the problem here. For sequence $s(s_1, s_2, \ldots, s_L)$, the model will give a “label” from the “label” space Ω to each position of the sequence. The label sequence can be described as $q(q_1, q_2, \ldots, q_L)$, where $q_i \in \Omega, i = 1,2, \ldots, L$. The task is to find the “label” sequence to maximize the joint probability Pr(q, s). Before describing the optimization algorithm, we’ll introduce some preliminaries:

(1) States of the model

Totally, there are 31 states in our model. They are grouped in 2 classes: coding

class Φ ={$Einit_{p0}^{+}$, $Einit_{p1}^{+}$, $Einit_{p2}^{+}$, $Esing^{+}$, $Eterm^{+}$, Ein_{p0}^{+}, Ein_{p1}^{+}, Ein_{p2}^{+}, $Einit^{-}$, $Esing^{-}$, $Eterm_{p0}^{-}$, $Eterm_{p1}^{-}$,$Eterm_{p2}^{-}$, Ein_{p0}^{-}, Ein_{p1}^{-}, Ein_{p2}^{-}} and non-coding states Ψ ={N, P^{+}, A^{+}, $Utr5^{+}$, $Utr3^{+}$, I_{p0}^{+}, I_{p1}^{+}, I_{p2}^{+}, P^{-}, A^{-}, $Utr5^{-}$, $Utr3^{-}$, I_{p0}^{-}, I_{p1}^{-}, I_{p2}^{-}}.

The states are described using forms as Xy_a^s:

X represents the types (E/exon, N/intergenic region, P/promoter, I/intron, A/polyA).

y gives additional position information in gene structures (Einit/initial exon, Esingl/single exon, Eterm/terminal exon, Ein/ internal exon, Utr5/5' UTR, Utr3/3' UTR).

a represents the "phase" (p0/phase 0, p1/phase 1, p2/phase 2). Phase of coding state is defined as the number of base(s) at the head of the exon which is integrated with base(s) from adjacent upstream exon to form a "codon" when translated to protein. Phase of non-coding state is defined as the same phase as the upstream exon's.

s represents the strands, plus strand or minus strand. All the states have a strand mark except the state N (intergenic region).

(2) Transitions

The transition probability from state x to state y is denoted as $T_{x,y}$, where $x, y \in \Omega$.

(3) Length distributions

Non-coding states can be very long and are assumed to have geometric length distributions. They are treated like states used in standard HMM in Viterbi algorithm. The lengths of coding states are limited compared with non-coding

states, and we use smoothed explicit distributions to describe the length distribution of coding states. Coding states are treated like states used in GHMM in Viterbi algorithm, i.e., at each position we search back a limited number of positions $\mathrm{L}_{\mathrm{max}_i}$ for state i instead of searching back over all previous positions.

(4) Emissions

The emission probabilities of coding states are based on an inhomogeneous 3-periodic fifth-order Markov model. Separate fifth-order Markov transition matrices are determined for hexamers ending at codon positions 1, 2 and 3, respectively. Therefore, the probability of state i to generate sequence $s_{a,b}$ is $E_i(s_{a,b}) = L(b-a+1) \cdot M_i(s_{a,b}), i \in \Phi$, where $L(x)$ is the probability of state i to generate a sequence with x nucleotide(s), and $M_i(x)$ is the probability of state i to generate the nucleotides x based on Markov model. On the other hand, the emission probabilities of non-coding states, $E_j(s_{a,b}), j \in \Psi$, are modeled using a homogeneous fifth-order Markov matrix. As described in the simplified method above, since state j ($j \in \Psi$) follows a geometric length distribution, the probability of state j to generate the sequence segment $s_{a,b+1}$ differs from that of generating $s_{a,b}$ by a factor, $p_j M_j(s_{b+1})$, independent of a, which is the basis that we can treat non-coding states as in standard HMM in Viterbi algorithm.

(5) Initial probabilities

We force sequence start from non-coding states, therefore the initial probabilities of coding states are set as a non-zero value MIN_VALUE. The emission probability of non-coding state j is represented as $\pi_j, j \in \Psi$.

Here, we first introduce our algorithm to predict the best gene structures (Top 1 Viterbi algorithm) based on this simplified method, and then introduce the extended algorithm (Top N Viterbi algorithm) with ability to predict the top N gene structures.

1) ALTSCAN Top 1 Viterbi algorithm

Top 1 Viterbi algorithm is based on a computation matrix $\Theta_{n \cdot L}$, where n is the state number of the model and L is the sequence length. In this matrix, variables $\Theta_n(m)$ are defined as the optimal joint probability of the subsequence $s_{1,m}$ which ends in state n at position m. For coding states, m must be the exactly ending position of state n. These variables can be calculated recursively as follows:

Initialization:

$$\Theta_n(1) = \begin{cases} \pi_n p_n M_n(s_{b+1}), & n \in \Psi; \\ MIN\_VALUE, & n \in \Phi. \end{cases}$$

Induction:

$$\Theta_n(m+1) = \begin{cases} max \begin{cases} \Theta_n(m) p_n M_n(s_{m+1}), \\ \max\limits_{j \in \Psi, \mathrm{j} \neq \mathrm{n}} \{\Theta_j(m)(1-p_j) T_{j,n} p_n M_n(s_{m+1})\}, \\ \max\limits_{i \in \Phi} \{\Theta_i(m)(T_{i,n} p_n M_n(s_{m+1})\}, \end{cases}, & n \in \Psi; \\ \max\limits_{\substack{j \in \Psi, \\ \mathrm{m+1-L}_{\mathrm{max}_n} \leq k \leq m}} \{\Theta_j(k)(1-p_j) T_{j,n} L(m-k+1) M_n(s_{k+1,m+1})\}, & n \in \Phi. \end{cases}$$

Termination:

$$\Theta_n(L+1) = max \begin{cases} \Theta_n(L), \\ \max\limits_{j \in \Psi, \mathrm{j} \neq \mathrm{n}} \{\Theta_j(L)(1-p_j) T_{j,n}\} \\ \max\limits_{i \in \Phi} \{\Theta_i(L)(T_{i,n})\}, \end{cases}, \qquad n \in \Psi$$

Finally, tracing back from the maximum probability from $\Theta_n(L+1),\ n \in \Psi$, we can obtain an optimal gene structure.

2) ALTSCAN Top N Viterbi algorithm

In order to find the top N probable gene structures, we need to keep the best N probabilities at each cell of the computation matrix. It is to say, the computation matrix is enlarged to $\Theta_{n \cdot L \cdot N}$ and variables $\Theta_n^t(m)$ are defined as the joint probability of the tth optimal parse of the subsequence $s_{1,m}$ which ends in state n at position m. These variables can be calculated recursively as follows:

Initialization:

$$\Theta_n^t(1) = \begin{cases} \pi_n p_n M_n(s_{b+1}), & \mathrm{t} = 1, n \in \Psi; \\ MIN\_VALUE, & \mathrm{t} = 2,3 \dots, \mathrm{N}, n \in \Psi \\ MIN\_VALUE, & n \in \Phi. \end{cases}$$

Induction:

$$\Theta_n^{1,2,\dots,N}(m+1) = \begin{cases} \max[N] \begin{cases} \Theta_n^t(m) p_n M_n(s_{m+1}), & t = 1,2,\dots,N; \\ \Theta_j^t(m)\left(1-p_j\right) T_{j,n} p_n M_n(s_{m+1}), & \\ \quad t = 1,2,\dots,N, j \in \Psi, \mathrm{j} \neq \mathrm{n}; & \\ \Theta_i^t(m)(T_{i,n} p_n M_n(s_{m+1}), & t = 1,2,\dots,N, i \in \Phi; \end{cases}, & n \in \Psi; \\ \max\limits_{\substack{j \in \Psi, \mathrm{t}=1,2,\dots,\mathrm{N} \\ \mathrm{m}+1-\mathrm{L}_{\max_n} \leq k \leq m}} [\mathrm{N}] \quad \{\Theta_j^t(k)\left(1-p_j\right) T_{j,n} L(m-k+1) M_n\left(s_{k+1,m+1}\right)\}, & n \in \Phi. \end{cases}$$

Termination:

$$\Theta_n^{1,2,\dots,N}(L+1) = \max[N] \begin{cases} \Theta_n^t(L), & t = 1,2,\dots,N; \\ \Theta_j^t(L)(1-p_j)T_{j,n}, & t = 1,2,\dots,N, j \in \Psi, \mathrm{j} \neq \mathrm{n}; \\ \Theta_i^t(L)(T_{i,n}), & t = 1,2,\dots,N, i \in \Phi; \end{cases} \quad , n \in \Psi$$

Finally, tracing back from the maximum N probability from $\Theta_n^t(L+1)$, $\mathrm{t} = 1,2,\dots,\mathrm{N}$, $n \in \Psi$, we can obtain top N optimal gene structures.

2.2 Training and prediction

Refseq annotation was cleaned by removing genes with in frame stop codons with eval-2.2.8[2]. 17,120 transcripts containing UTR regions (5' UTR and 3'UTR) were

used for training. Although ALTSCAN used an extended multi-layer Viterbi method, it was trained only at the first layer (TOP 1).

ALTSCAN needs a computation space of $\Theta_{n \cdot L \cdot N}$ as described above, therefore it is hard to predict gene structures directly on the whole genome/chromosomes. In practice, the candidate gene regions were extracted as input of ALTSCAN. GENCODE basic V12 genes, which were derived from HAVANA manual annotation process and Ensembl automatic annotation pipeline, and RefSeq genes[3] were used as the known genes. SIB genes[4], having support evidence of at least one GenBank full length RNA sequence, one RefSeq RNA, or one spliced EST, were used to create regions with mRNA or EST evidences. In addition, genomic regions with NSCAN genes[5], using multiple-genome alignment information for prediction, were also included in the candidate regions. Totally, 33,480 sequences with a padding length of 5,000 bp at both ends of genes were picked out. In our work, 31,140 (93%) sequences with lengths shorter than 600k bp were predicted for the best 250 gene structures and the remained 2,340 sequences were predicted for only the top 100 gene structures. For all predicted transcripts, since the protein-coding regions were our focus, only one copy of those transcripts with a consistent coding region but different UTR regions was remained. Totally we obtained 8,019,000 raw transcripts. 1,730,400 transcripts (21.6% of all raw transcripts) had unique protein-coding sequences. After this, 1,409,616 transcripts (81.46% of 1,730,400), which didn’t have complete coding regions, were also filtered out. Finally, 320,784 transcripts with complete coding regions from 33,945 genes made up the ALTSCAN dataset.

2.3 Evaluation

Refseq genes and GENCODE genes were combined as KNOWN dataset, which was used as a gold standard. Transcripts were merged to genes if they overlapped and located on the same strand. KNOWN dataset had 55,177 transcripts from 19,924 genes and 9,780 transcripts from 8,325 genes in ALTSCAN dataset were consistent

with the KNOWN dataset. Therefore ALTSCAN's gene level sensitivity was 8,325/19,924=41.8% and ALTSCAN's transcript level sensitivity was 9,780/55,177=17.8%. ALTSCAN's primary purpose was to exhaust all the reasonable protein-coding gene structures, so we didn't evaluate its specificity.

As described above, ALTSCAN used an extended multi-layer Viterbi algorithm. Here we showed this extended method indeed helped to find more transcripts. To see how much we were benefited from the predicted suboptimal structures, the KNOWN dataset was used as a gold standard. As shown in Figure S2, with a traditional Viterbi algorithm for GHMM, which meant only the most probable structure was predicted for one input sequence (see Methods for more details), only 1,509 transcripts were found to be consistent with KNOWN transcripts. Overall, 84.6% of ALTSCAN transcripts consistent with KNOWN transcripts came from suboptimal paths, demonstrating that it was effective to predict alternative transcripts by including more suboptimal paths.

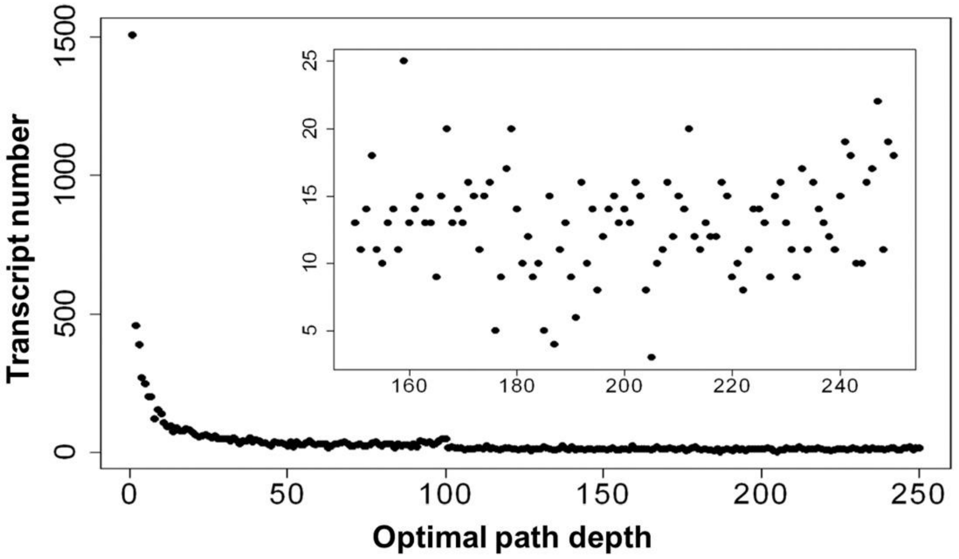

Figure S2. The number of predicted transcripts consistent with KNOWN dataset vs. the depth of optimal paths. X axis shows the depths of ALTSCAN predictions and Y axis shows the number of transcripts predicted at a specific depth and consistent with KNOWN dataset. The enlarged figure in the middle shows transcript number distribution after the depth 150. The gap at the depth 100 is because that all

transcripts have predictions within depth 100 and those relatively long transcripts (length > 600k bp) don't carry out prediction at depths over 100.

We further compared ALTSCAN with 4 ab initio predictors: 1)ALTSCAN, 2)Genscan[1], 3)Geneid[6] and 4)AUGUSTUS[7] (AUGUSTUS_noRNA) and 7 predictors using RNA-seq data: 1)AUGUSTUS[8] (AUGUSTUS_RNA, corresponding to AUGUSTUS_all in RGASP), 2)Exonerate[9] (corresponding to Exonerate_all in RAGSP), 3)mGene[10], 4)mTim, 5)NextGeneid, 6)Transomics (Corresponding to Transomics_all in RGASP) and 7)Tromer[11]) based on the KNOWN annotation. Predictions from AUGUSTUS_no_RNA and all predictors using RNA-seq data were downloaded from https://www.ebi.ac.uk/arrayexpress/experiments/E-MTAB-1730/files. Details of the predictors using RNA-seq data and the generation of their predictions can be found in RGASP[12,13]. The evaluation on gene-, transcript- and exon-level was achieved with the tool RGASP.jar provided by RGASP.

2.4 Deficiency

ALTSCAN was used to find transcripts as many as possible, while it had several deficiencies. First, we applied ALTSCAN to candidate gene regions instead of the whole genome/chromosomes. Although we considered known protein-coding gene regions, predicted gene regions and regions only having EST supports, we might still miss potential protein-coding gene regions. Second, N=100 or even N=250 might not be deep enough. As shown in Figure S2, there were still 14 transcripts found at each depth from 150 to 250, and this number didn't seem to go down. In addition, ALTSCAN didn't take rare splice signal, like GC-AG, into consideration. These deficiencies hindered ALTSCAN's exhausting protein-coding gene structures.

3 Dataset construction

In order to construct a comprehensive transcript dataset, we first collected transcripts from the GENCODE and RefSeq database. Then GENCODE and RefSeq transcripts

were combined and filtered. Transcripts sharing the same coding regions, having internal stop codons or short introns (<20bp) were removed. After filtering, the refined dataset was named KNOWN dataset, which represented all currently known transcripts. Then ALTSCAN dataset and KNOWN dataset were merged with redundant ones removed to form a MIXTURE dataset.

4 RNA-seq validation

4.1 RNA-seq data information

We collected 50 RNA-seq runs from the Illumina Human BodyMap2 project and ENCODE project. They were utilized to validate transcripts in the MIXTURE dataset (Table S1). Different runs of a biological sample were merged together as a single dataset. Finally, we obtained 26 datasets, which could be classified into to 3 groups: GROUP I, data sequenced from a single tissue from the Illumina Human BodyMap2 project; GROUP II, data sequenced from a mixture of 16 tissues from Illumina Human BodyMap2 project; and GROUP III, data from ENCODE project.

Table S1. RNA-seq data information

GROUP ID	Dataset ID	Run ID	BioSample ID	Source	RNA Extract	Paired/Single read lengths	SRA run accession	UCSC Accession	Karyotype	Tissue	Description
I	1	1	HCT20158	Illumina Human BodyMap2	PolyA+	1x75	ERR030888		cancer	adipose	
		2	HCT20158	Illumina Human BodyMap2	PolyA+	2x50	ERR030880		cancer	adipose	
	2	3	HCT20159	Illumina Human BodyMap2	PolyA+	1x75	ERR030889		cancer	adrenal	
		4	HCT20159	Illumina Human BodyMap2	PolyA+	2x50	ERR030881		cancer	adrenal	
	3	5	HCT20160	Illumina Human BodyMap2	PolyA+	1x75	ERR030890		cancer	brain	
		6	HCT20160	Illumina Human BodyMap2	PolyA+	2x50	ERR030882		cancer	brain	
	4	7	HCT20161	Illumina Human BodyMap2	PolyA+	1x75	ERR030891		cancer	breast	
		8	HCT20161	Illumina Human BodyMap2	PolyA+	2x50	ERR030883		cancer	breast	
	5	9	HCT20162	Illumina Human BodyMap2	PolyA+	1x75	ERR030892		cancer	colon	
		10	HCT20162	Illumina Human BodyMap2	PolyA+	2x50	ERR030884		cancer	colon	
	6	11	HCT20143	Illumina Human BodyMap2	PolyA+	1x75	ERR030894		cancer	heart	
		12	HCT20143	Illumina Human BodyMap2	PolyA+	2x50	ERR030886		cancer	heart	
	7	13	HCT20142	Illumina Human BodyMap2	PolyA+	1x75	ERR030893		cancer	kidney	
		14	HCT20142	Illumina Human BodyMap2	PolyA+	2x50	ERR030885		cancer	kidney	
	8	15	HCT20144	Illumina Human BodyMap2	PolyA+	1x75	ERR030895		cancer	liver	
		16	HCT20144	Illumina Human BodyMap2	PolyA+	2x50	ERR030887		cancer	liver	
	9	17	HCT20145	Illumina Human BodyMap2	PolyA+	1x75	ERR030896		cancer	lung	
		18	HCT20145	Illumina Human BodyMap2	PolyA+	2x50	ERR030879		cancer	lung	
	10	19	HCT20146	Illumina Human BodyMap2	PolyA+	1x75	ERR030897		cancer	lymph_node	
		20	HCT20146	Illumina Human BodyMap2	PolyA+	2x50	ERR030878		cancer	lymph_node	
	11	21	HCT20150	Illumina Human BodyMap2	PolyA+	1x75	ERR030901		cancer	ovary	
		22	HCT20150	Illumina Human BodyMap2	PolyA+	2x50	ERR030874		cancer	ovary	
	12	23	HCT20147	Illumina Human BodyMap2	PolyA+	1x75	ERR030898		cancer	prostate	
		24	HCT20147	Illumina Human BodyMap2	PolyA+	2x50	ERR030877		cancer	prostate	
	13	25	HCT20148	Illumina Human BodyMap2	PolyA+	1x75	ERR030899		cancer	skeletal_muscle	
		26	HCT20148	Illumina Human BodyMap2	PolyA+	2x50	ERR030876		cancer	skeletal_muscle	
	14	27	HCT20151	Illumina Human BodyMap2	PolyA+	1x75	ERR030902		cancer	testes	

		28	HCT20151	Illumina Human BodyMap2	PolyA+	2x50	ERR030873		cancer	testes	
	15	29	HCT20152	Illumina Human BodyMap2	PolyA+	1x75	ERR030903		cancer	thyroid	
		30	HCT20152	Illumina Human BodyMap2	PolyA+	2x50	ERR030872		cancer	thyroid	
	16	31	HCT20149	Illumina Human BodyMap2	PolyA+	1x75	ERR030900		cancer	white_blood_cell	
		32	HCT20149	Illumina Human BodyMap2	PolyA+	2x50	ERR030875		cancer	white_blood_cell	
Ⅱ	17	33	HCT20170	Illumina Human BodyMap2	PolyA+	1x100	ERR030856		cancer	16 tissues	
		34	HCT20170	Illumina Human BodyMap2	PolyA+	1x100	ERR030857		cancer	16 tissues	
		35	HCT20170	Illumina Human BodyMap2	PolyA+	1x100	ERR030858		cancer	16 tissues	
		36	HCT20170	Illumina Human BodyMap2	PolyA+	1x100	ERR030864		cancer	16 tissues	
		37	HCT20170	Illumina Human BodyMap2	PolyA+	1x100	ERR030865		cancer	16 tissues	
	18	38	HCT20172	Illumina Human BodyMap2	PolyA+	1x100	ERR030859		cancer	16 tissues	
		39	HCT20172	Illumina Human BodyMap2	PolyA+	1x100	ERR030860		cancer	16 tissues	
		40	HCT20172	Illumina Human BodyMap2	PolyA+	1x100	ERR030861		cancer	16 tissues	
		41	HCT20172	Illumina Human BodyMap2	PolyA+	1x100	ERR030866		cancer	16 tissues	
		42	HCT20172	Illumina Human BodyMap2	PolyA+	1x100	ERR030867		cancer	16 tissues	
Ⅲ	19	43	GSM758561	ENCODE	longPolyA+	2x76		wgEncodeEH000144	normal	lung	AG04450,fetal lung fibroblast
	20	44	GSM758562	ENCODE	longPolyA+	2x76		wgEncodeEH000145	normal	skin	BJ, skin fibroblast
	21	45	GSM758559	ENCODE	longPolyA+	2x76		wgEncodeEH000146	normal	blood	GM12878, B-lymphocyte, lymphoblastoid
	22	46	GSM765401	ENCODE	longPolyA+	2x76		wgEncodeEH000167	normal	skin	NHEK, epidermal keratinocytes
	23	47	GSM758564	ENCODE	longPolyA+	2x76		wgEncodeEH000143	cancer	epithelium	A549, epithelial cell line derived from a lung carcinoma tissue
	24	48	GSM765402	ENCODE	longPolyA+	2x76		wgEncodeEH000173	cancer	cervix	HeLa-S3, cervical carcinoma
	25	49	GSM758575	ENCODE	longPolyA+	2x76		wgEncodeEH000160	cancer	liver	HepG2,hepatocellular carcinoma
	26	50	GSM765405	ENCODE	longPolyA+	2x76		wgEncodeEH000163	cancer	blood	K562,leukemia

4.2 Validation strategies

Quality control of RNA-seq data were automatically processed using the NGSQC program with default parameters[14]. Coding sequences from MIXTURE transcripts were extracted with 100nts upstream start codons and 100nts downstream stop codons. These coding fragments formed the mature transcript dataset. High quality reads were mapped to mature transcript dataset using Bowtie [15] with the option "-l 28 -n 2 -a --best".

We created a pipeline to validate candidate transcripts. For each transcript, we checked the coverage of all bases (full transcript coverage) and the coverage of all splice junctions (junction coverage). A base was covered if there was at least one read mapped to the position. We focused on validation of the splice sites of these transcripts. This wasn't to say transcription was not important. To balance this, we give the rule that all the bases of a transcript should be covered at least once, which was quite loose, but effectively showed the transcription. A splicing junction site was covered if and only if at least M read(s) covered both sides of the adjacent exons with no less than L nt(s). Therefore, it was possible that a transcript was fully covered but not all the junctions were covered and vice versa. We developed multiple levels of validation criteria. For all transcripts (both single exon transcripts and multi-exon transcripts), full transcript coverage should be 100% in order to be called validated. For multi-exon transcripts the junction coverage should be 100% as well. In this step, two strategies (the standard strategy and the stringent strategy) were used to check the junction coverage. In standard strategy, M was set to be 1 and L was set to be 10. In the stringent strategy, values of M and L depended on the RNA-seq data groups. We described how this was done in following parameter selection part.

In addition, additional rules were used to validate novel transcripts (in ALTSCAN but not in KNOWN dataset) in three levels, i.e. VHC (validation with high confidence) VMC (validation with median confidence) and VLC (validation with low confidence). Multi-exon transcripts validated in stringent strategy with one or more novel internal

splice junction sites (NIJ filter, see following and Figure S3 for details) were described as VHC transcripts; multi-exon transcripts validated in standard strategy with one or more novel internal splice junction sites were described as VMC transcripts; and multi-exon transcripts validated in standard strategy without novel internal splice junction sites, together with validated single-exon transcripts were described as VLC transcripts.

As shown in Figure S3, there were two situations that validated transcripts had no novel internal splice junctions. The first situation was an alternative translational start sites and the second situation was an intron retention in the KNOWN transcripts. These transcripts were also put into the VLC datasets. The remained transcripts with novel internal junctions would be put into the VHC transcripts.

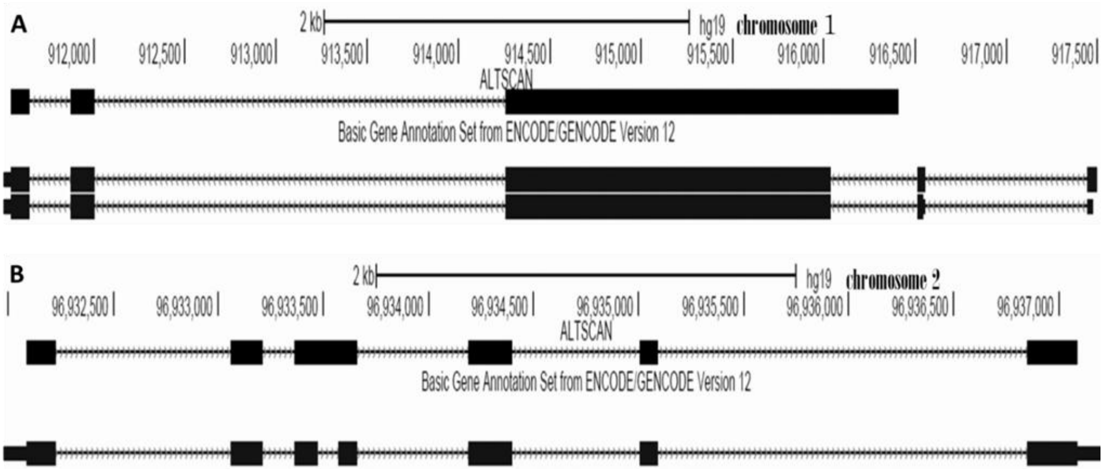

Figure S3. NIJ filter, illustrations of validated multi-exon transcripts without novel internal splice junction sites. A. an ALTSCAN transcript with an alternative translational start sites compared with GENCODE transcripts. But the two internal splice junctions are already included in GENCODE transcripts. **B.** an ALTSCAN transcript with an AS event of intron retention compared with GENCODE transcripts. But all the internal splice junctions are already included in GENCODE transcripts.

4.3 Parameter selection

Here we showed how we selected parameter *M* and *L* for stringent strategy. The sequencing depth and read length may impact on the selection of *M* and *L*. Therefore different datasets might need different parameters. Consider that different datasets from the same data group had the same sequencing lengths and the data size was quite

close, and to be comparable between validations from different datasets, we decided using the same parameters for datasets from a same group.

In order to select reasonable values for *M* and *L*, we validated a datasets with different combination of *M* and *L*. Then we checked the number of VHC transcripts we obtained under each combination. When *L* was fixed, it was clear that the number of VHC transcripts declined if *M* got larger. When *M* was small, many spurious transcripts together with some very-low-expression transcripts would be validated. A reasonable value of *L* would be obtained when the remained proportion of VHC transcripts was stable. Meanwhile when, when *M* was fixed, *L* should be selected similarly. Finally, L=8, M=5 were used for validation with GROUP I datasets; M =8, L=8 were used for validation with GROUP Ⅱ datasets; and M=5, L=9 were used for validation with GROUP Ⅲ datasets (Figure S4).

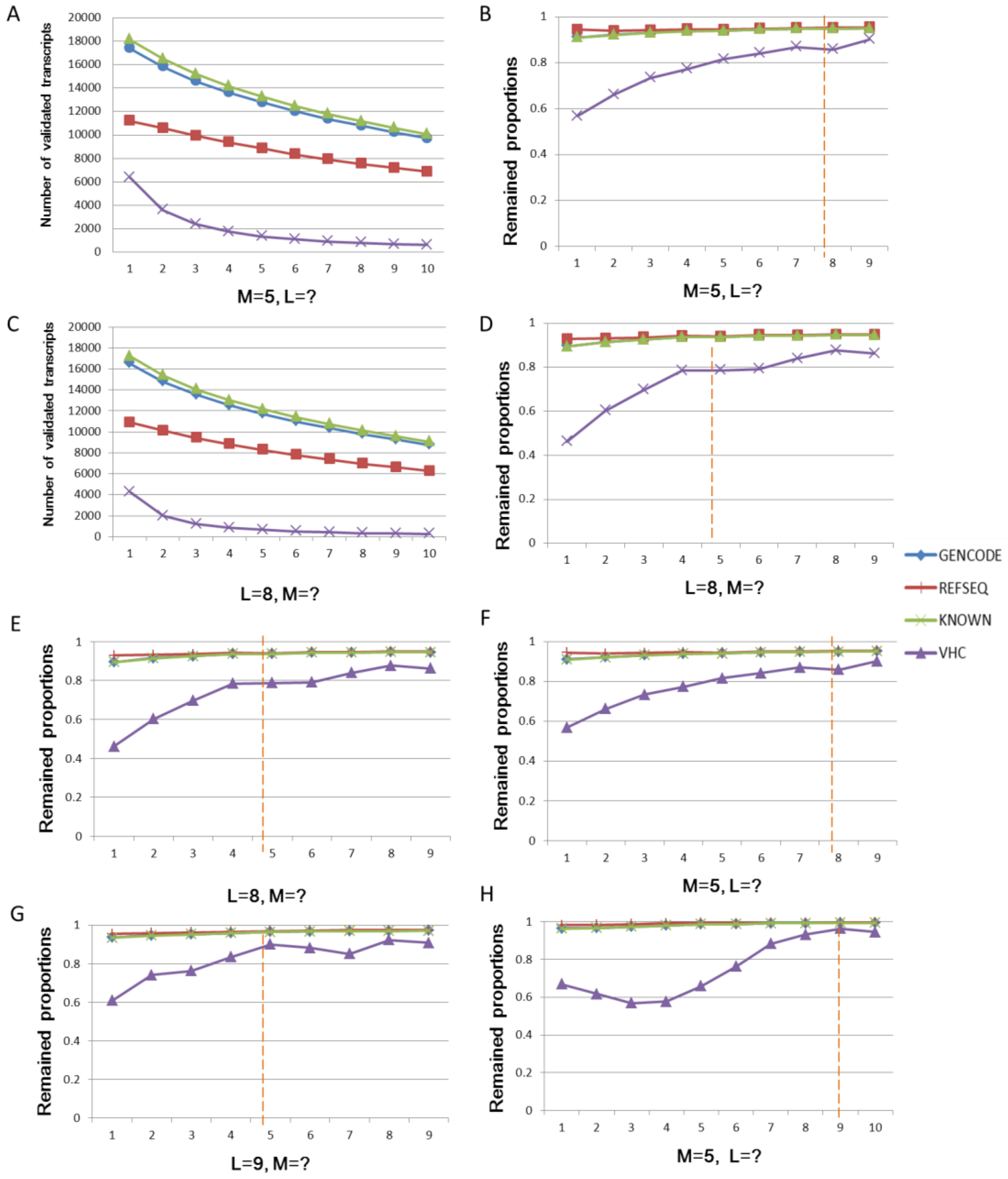

Figure S4. Parameter selection in stringent strategy. A-D showed parameter selection for Dataset 17 (Sample ID: HCT20170). This was an example to show how we select reasonable values for M and L. First, we checked the numbers of VHC transcripts for each combination of M=1,2,…,10 and L=1,2,…,10. Second, we fixed M (or L), and checked the variation trend of number of VHC transcripts as L (or M) got larger. **A** showed the number of VHC transcripts when M was fixed (M=8) and L changed (L=1,2,…,10). **C** showed the number of VHC transcripts when L was fixed (L=8) and M changed (M=1,2,…,10). Next, we still fixed M (or L) and examined the remained proportions from L=i to L=i+1 (or from M=i to M=i+1), i=1,2,…,9. **B** showed the remained proportions when M was fixed (M=8) and L changed. **D** showed the remained proportions when L was fixed (M=8) and M changed. Finally, L=8 and M=8 were selected for this dataset. We give two examples of parameter selection of datasets from Group I and III. **E** and **F** showed parameter selection for Dataset 3

(Sample ID: HCT20160, from Group I). **G** and **H** showed parameter selection for Dataset 20 (Sample ID: GSM758562, from Group III). The remained proportions for I was calculated as N(i)/N(i+1), where N(i) was the number of VHC transcripts under parameter i. The red dashed line showed the value that the remained proportion was stable.

4.4 Validation landscape

We validated MIXTURE transcripts with the pipeline and parameters described above. The numbers of validated transcripts from each dataset were shown in Table S2. By comparing validation of KNOWN and novel transcripts (Table S2), we showed that novel transcripts have much higher levels of tissue-specific expression than KNOWN transcripts do (Table S3).

Table S2. Transcripts validated from each dataset.

Dataset	Transcript number			
	KNOWN_standard	KNOWN_stringent	VMC	VHC
Dataset 1	15,237	12,550	3,832	1,163
Dataset 2	15,893	12,744	4,286	1,142
Dataset 3	16,742	13,009	3,316	830
Dataset 4	14,883	11,944	3,462	1,012
Dataset 5	13,165	11,321	3,184	1,046
Dataset 6	17,593	14,494	3,212	793
Dataset 7	16,168	13,953	4,144	1,175
Dataset 8	12,751	10,773	3,163	1,041
Dataset 9	14,736	13,452	3,094	1,137
Dataset 10	13,667	12,597	2,792	944
Dataset 11	18,112	14,558	3,584	984
Dataset 12	17,373	15,624	3,618	1,168
Dataset 13	9,710	9,062	2,044	731
Dataset 14	20,611	17,477	4,130	1,200
Dataset 15	17,838	14,612	3,715	1,036
Dataset 16	14,031	12,868	3,535	1,291
Dataset 17	19,102	15,403	1,059	286
Dataset 18	19,187	15,550	1,094	329
Dataset 19	14,723	11,866	787	223
Dataset 20	19,740	16,286	1,487	431
Dataset 21	21,144	17,297	1,573	448
Dataset 22	12,422	10,138	655	218
Dataset 23	17,048	14,167	986	356

Dataset 24	16,108	13,141	1,032	310
Dataset 25	27,850	19,522	18,836	6,131
Dataset 26	26,894	20,333	18,009	7,530

Table S3. Transcript numbers grouped by number of validated datasets.

Dataset	Transcript number			
number	KNOWN_standard	KNOWN_stringent	VMC	VHC
1	4,669	5,429	11,695	4,747
2	3,676	3,422	8,523	3,958
3	3,372	2,685	3,626	1,021
4	2,420	1,873	2,021	540
5	1,988	1,642	1,358	283
6	1,709	1,336	939	200
7	1,465	1,172	713	162
8	1,331	1,094	573	121
9	1,256	979	425	94
10	1,174	953	340	81
11	1,100	788	310	90
12	933	760	231	78
13	1,002	789	182	65
14	937	741	193	53
15	919	713	152	40
16	904	725	128	41
17	904	696	99	44
18	868	700	106	59
19	849	759	32	20
20	865	684	30	14
21	922	729	32	5
22	1,006	821	30	9
23	974	803	15	8
24	1,110	928	23	16
25	1,422	1,177	22	12
26	3,022	2,639	21	11
Total	40,797	35,037	31,819	11,772

5 PCR validation

We randomly designed primers flanking novel splice sites for 88 VMC transcripts using Primer3. We ensured that primers were uniquely (at least 2 base mismatches for suboptimal alignments) mapped to human transcriptome or genome (hg19). We also ensured the product sizes were from 250 bps to 500 bps. Primer sequences were shown in Table S4. 88 primers flanking novel splice sites and 8 primers flanking known splice sites from house-keeping genes were selected (Supplemental Table S4).

Table S4. Primer lists.

id	forward primer	reverse primer
h01	CTCAGTCCCTTTCAGTACATGGAT	TAAAGACTCTGTGACTTGTGCTGGT
h02	TTCTGAAGCAGTTCAATTGTAAGA	AGAGAATTATTCTGGATAAGTTGAA
h03	GACTCTTCAAGTTTTCTGTTCCAA	CTAAACCTTTCTGGAAATGAATTGA
h04	GCCGTGTTGTAAAATTATTCAGAA	ACAGTTATACCAAACTCACATGTTT
h05	GATCAAAGTCATTAAATCTGTTCT	TAGAAATTGAAAGGCAAAAACTAG
h06	CCTTTCTGTTTCTGATTTCTTCTAC	TTGGAAAAACAACATTGATCCATAA
h07	CATGTGTCATAGTCAATGTCTGTC	CTCTCCACTTCTTTGTCAATGGGATG
h08	TATACCATCAGATACTTCTCTGGA	AATTTCAGATTATTTCAGGAAACAG
n01	TGACGATCTGATTGTCATCCAGGA	CCTCGCAGGATGGTAAACTTATCAT
n02	GATTTCTTTTGACTAGAATCTTGT	GTACACAGTTGTCAAAGGCATCATC
n03	CTCCAGACATTTCCTAAGGAGTTC	CAATATTGTCCAGATGAAGGACGGA
n04	GAATTTTCTGAGCAATAACGAAC	AAAATCGAAAGACTAAAATCAGAG
n05	GATGATAAGTCTGGAAGATCATA	ATACATGTTTTCCCTAGAAGAGAAT
n06	GAAGGATTCGAGAAGGAGTGATA	CCAAGAATGCTGATGGGACTATATG
n07	TAGATGAAGTCATTATGGTACTG	CTGATGTGTATGACTTCTTCCTCCAG
n08	CTTGCTTTTTAGGTTTTTAGCTTG	TAAGACAATCTCTCAAGAATTTCTT
n09	GAATATGTAGAAAATGAATCCAT	TACTTATGAGGTCAATTGGAAATAA
n10	TTTTTCCTTTTGGTGATATAGAAA	AATTAAACCTGTATCAAGATGGGAA
n11	ATGGAATTGAAGAAGTAATAGCC	CATCATTCTCATCAGCTTTTCCTGGT
n12	ACTGAATGAATTATCAATACTCTC	CTATTCTTCAGACATGCACATTTCCA
n13	CAATTATTTTCTGCTTTGCATCAA	TATGAACAAGAAATTACTAAACTGA
n14	TCTTAGTTACAGTTATTCCTTTGC	GCTTGATCTCTATAGGCAAATGGAT
n15	CATCAAGTCTAAATAGATGTCAG	AGTGAAGAAAATATTGAATTTTGGA
n16	TTGTAAGATAGTTGTTCGTGTCCT	CAGATGAAGTTTTAGCAGAAGCAAA
n17	ACTATAATATTGACTTGGTGTTAG	AAAGATTGATGGAAATATGTGAAAG
n18	TTCGTAGTCTTTGTATGTGGTATA	CAATTTTAGAAGATGCTATACCCTC
n19	CAAAAATAAGTTCTTTTGTTCAGC	CAGTCCTTGCTGTAAAAACTGTCAG
n20	CATCAAAGACTTCTTCTGACTCCT	GTTTTCAAACCCTTCATTGATCTCGA
n21	TCTCAGCTCAGTAACCAGCTTATT	GAATAAGTATGGGATGGTAGCACAA
n22	CTCTTATGGTTATTCTTCCATAGT	ATTAGTAAGCAACTCTCTGAAGAAT

n23	TTCTTTGGGATAAATTCATATACC	TGCTTTTAAATACTTCTATAGAACA
n24	AAGAGAAATCCATAGGTTGTTTG	GTACCCGAGGATTAATAAAGAATCT
n25	GAGTTTTAGAATCAGGGCGTAAC	CTGTTAAACACATTGGATATGATGA
n26	ATGTTTTTGAAAGTTGTCACCAGT	CAGGAAAACTTTGAAGCCTTCATGA
n27	TATATTCCCTGTTTTCTGGAGTAT	GTATTGCTATTAAGGAGTCAGCAAA
n28	GTCCAAGATACCAACAGACAGTG	TCTTATGTCCTACAAAGCTGAAGAT
n29	GTAGTATCTATGAACTGTTTCAAG	CTTTTTGATGTGGTTTATTGAAACTG
n30	CACATTTATCAGATGCCTTTTCAT	GATTGAATAAGGAAATAATGATGGA
n31	CTAGGTGTAGGATCCCATATTTTT	CTAAAGCTGGAGAACTAAAAGTCGT
n32	ATATTGCAAATATCTTCTTCTGAA	ATATAGATTGAAGTTTAGCGCTGAT
n33	TTCATATTTTTCTGTTTTTGCTAAT	CTAAGTATGGTCTCATTTATCATGCT
n34	CTCTCTTCACTCCTGTTTTAAACTT	TCATTATTTAGCAATGTTGTTACCAA
n35	ATAATTCGCAAACATAAAGGGAT	TGTAAATATTTATCCATCTATCAACC
n36	TAATTTTTGAAGACTGATCTGCTA	CAGATATTGCAGTAATCATGTACAC
n37	CATGTCCAAAAGGTGAAATAAAG	ATAAACAGCCTATTCCTAACTGATC
n38	CATCAACAGCATGGAGAAGAAGA	CTACAACTCTTTCCACAACAATGTCT
n39	CTTCAGTTCATCTTGCTTTTCAAA	TTTGAATGCATGTATTCACTGCTTGA
n40	CGACTTAATTCCTGTTGAAGACTG	GGAAATGAAAGAAGAAGAAATTGC
n41	GAAGTCTTTTTGATGTTCCTTTAT	GAAGAGAACCCAAGAAATCAATTA
n42	CTTGGAACACTTAAAGGACTATG	TCCATAAGGAATGTAAAAATATGAG
n43	GAGTAGCGAATAAATGCTCTTCCT	TGACAGAACTAAGCAAAGAATTTCA
n44	CTATGATGTAAAAGCTATCAGCCT	GGGGAACTTATGACATTTTAGTAAC
n45	CAAGTGAATTTATGTCAGCTGTTC	TATGTGAAACAGCCTTTACCTGATG
n46	CTGATCCAATTCAGAGTAGACTGT	AAGCAGATGAAGTACTTAGAGCAGC
n47	CTCTTCAAACCTATTCCTTCCATT	GTTTCTGAGTCCCTTCAACATGATCC
n48	TAATATCCCTGTAGAAGTCACAAT	TAAAGGAAGAGATATTTACACCTTT
n49	CAGTATACAGGAAGATGCAGTTG	CTTTGGAGTCAGCATCTACCTAATC
n50	CTCGATGACATCAAGAATCTCTTC	TTCATCTATGAATTCGAGCACTTCA
n51	TTCTTGATGCTGGTGATTTCACTA	GAGAAAGAATCAACGGAAGACTAC
n52	GAAAAGACTTCATCATCTTATATG	AAGATCTCCAGTATGTGAAGATAGA
n53	TCTCATTTAAATATCTACTATACC	TTAATTCAGTGCACAAATGAGATGA
n54	GTATGTTACAGTAATTTTTCTTGG	CTACAAACCACAGAGGAATCTTTTA
n55	CAAAAGATGGCAATCCCTTCATT	GTTTAATGATCACCTCTGCTGCAATC
n56	AAAGTCTTTATTCCCAATCTCAGC	AGAGGAAAAAGCCTATGAGATCAT
n57	ATCACAACTATCGTAGCTCTCTAT	CAGCTTCATCACAGAGTCCTATCAG
n58	TTGGAGCCCAAACAATTGCTAGA	CTATGTAAGCTGTACTTCCGGGAAC
n59	CTTAGCTACTTTACACAGTCCCAT	ATATTTGCCATCACAGTTGCTACAA
n60	GACACAGTTATCTTCTTCATGGTT	GAAAATGAGTTTTCTTTAAGGCCAA
n61	TATCTATATGGCGATTAGAAGTTG	AAAAGAACAGTTTATTCCACCATAT
n62	CTGATTGACTTGAATGAAGAGTA	GCCATAATGACCTATTGTGTAAGAA
n63	CACACTCACACGACTGATAGTAG	CTCAAATATATGACCCCTGTGATTCT
n64	GTATCCGAGAAGTGTCTTTGTGAT	AATAAAATTGAAGACTGGCTGCAGG
n65	CTATGAGATAAGCAACAATTGAG	GAAAACTCATATTGATGATTACAGC
n66	CATGAAGTAGATTTTGTCTTGTTT	ATGATATGATGCCTAAATACCTCAA
n67	GAACAAGTATGTCCGAAATGTAC	CTAAAGAACTTTGGGATCAGTTTCA
n68	GACAACTAAAACCCTGATTGTCT	GCACAGTCCTTATCAATGTTGAAGA
n69	GTCTGTTATTTTTATTGATAATCT	GTGATCAGTGATGATACTTTTGAGT
n70	GTCTTCATCTGTGACAATAAAAGC	CATTGTCTCTCTTACTGACTGTCACA
n71	GAATCGGATGCCAAGCTTGTAAT	CCTGTGGATAGAGGAAAAAGGAAC

n72	AAAACCTACAGAAGATATGTAGG	GAACATTGAAGAGCAGTACAAAAA
n73	GGATGTAAACAACGTCAAAGAAG	AATGTCTCCCAGAAGATGATTGAGA
n74	CTTGGTGAACACAATAGAGTTGG	CCAATGGGAAATACATCACCAAATC
n75	ATCATTTTTCCCCAGTGGCTCATC	TGGAGAAGTACACGATCTCACAAGA
n76	AAAATCCCGAAACTTGGTGTCCTT	GTTCTACTTGAAGAACCTGATGATG
n77	GAGTGTAATTGTTGATGAAAATC	TAAGGGAAGAAAAGATTTGGATGAT
n78	GTAGATAATTTTTCTACCAGGGAT	CTTTAACGCCAAAAGGAAAAAGAA
n79	AAATATTGACGATAAAGCTCTTCT	GATCTTAGAGACTTTCGTGATCTAA
n80	GATAGAGGACACAATCTGGCTAA	CTCATAGAACGTCTCTTAGTTGATTA
n81	CAAAGATGAGCCTCTGATTTTCGA	AAAATGTCAAAGCCAAAATCCAAG
n82	TTTTTATATTGTGCTTGTTCCTGTA	AAAGCAGAAAGAGTATTTGATGATC
n83	TTCATTACACTTTATTTCTTTACCT	CTGGACATTGAATATTTTACTGATG
n84	TTCATCAGTAATACAAATGTGGTC	AAGATCTGATGATGATGATACAAAT
n85	GATGAAGTATATGCATCTGTGAA	CTTCTCATGAAAATTCAACGGCTAC
n86	CAAAACTTCCAACACTAATTTATT	GCTAGCAAGATTAATAAAGAAACTA
n87	GCTTGATTTATCTTTGATGTCTTT	AGTTCTAAAGATGGAAGAAAATTTT
n88	TTTATCTCCTTTAGCATCCCCTTC	CAAAAGACTAGATCAAGTAGAAGT

5.2 Real-time PCR experiment

Real-time PCR validation was carried on in a range of tissues and cell lines. 20 cDNA samples from human haematopoietic cell lines, 20 cDNA samples from different human tissues and cDNA from 8 acute myeloid leukemia (AML) samples from patients were used for analysis of gene expression and sequencing (Supplementary Table S5).

Specific target amplification (STA) was used to increase the number of copies of target cDNA. A 500 nM primer mixture (10x) was prepared by pooling 1 μL aliquots of all the primer pairs (100 μM) to be included in the STA reaction and the final volume adjusted to 100 μL. The pre-mix for the STA reaction was prepared by mixing 2.5 μL 2xTaqMan PreAmp Master Mix (Applied Biosystems), 0.5 μL 500nM (10x) pooled primer mix, and water to a total of 5 μL. 3.75 μL of the STA pre-mix was aliquoted for each sample (40 or 48), and added 2 μL cDNA (~1 ng/μL). The samples were then amplified (95 ℃ for 10 min, (95 ℃ for 15 sec, 60 ℃ for 4 min) x 12/16 (13/17) cycles), 4 ℃). Number of cycles was set to be 12 for the cell lines and

AML cDNA samples, and 16 for the human tissue cDNA samples. The STA samples were diluted 1:10 before use on the Dynamic Arrays.

The Dynamic Arrays were run according to the manufacturer's protocol. Briefly, sample pre-mix was prepared using 2xSsoFast EvaGreen Supermix with Low ROX (Bio-Rad, PN 172-5211), and 20x DNA binding Dye Sample Loading Reagent (Fluidigm, PN 100-3738), both to a final concentration of 1x. 3.3 µL of this mix was added to 2.7 µL of each diluted STA sample (a total of 48 reactions). The assay mix was prepared by mixing 25 µL 2xAssay Loading Reagent, 2.2 µL of 100 µM mix of the combined forward and reverse primers for each assay, and PCR grade H2O to the final volume of 50 µL (stock). The Dynamic Array IFC were primed by loading control line fluid into each accumulator on the chip, before lading the chip into the IFC controller MX (48.48, and the Prime (113x) script) or the IFC controller HX (96.96 and the Prime (136x) script). After priming, 5 µL of each assay and sample were added to their respective inlets on the chip. Using the IFC controller software, the Load Mix (113x) script (48.48) or Load Mix (136x) script were run.

The chips were then run on the BioMark HD using standard conditions on the Data Collection Software: 70 ℃ for 40 min, 60 ℃ 30s, 95 ℃ for 60s, (96 ℃ for 5s, 60 ℃ for 20s) x 30 cycles, 60 ℃ for 3s, hold at 60-95 ℃.

Barcodes were attached according to the manual. Briefly, a sample pre-mix solution was prepared, containing 1x FastStart High Fidelity Reaction Buffer without MgCl2 (Roche), 4.5 mM MgCl2 (Roche), 5% DMSO (Roche), 200 µM ea PCR Grade Nucleotide mix (Roche), 0.05 U/µL FastStart High Fidelity Enzyme Blend (Roche), PCR grade H2O to 15 µL pr. reaction. The harvested PCR products were diluted 1:100, and 1 µL of diluted amplicons was added to 15 µL sample pre mix and 4 µL Access Array Barcode Library for Illumina Sequencers 384 (Single Directions). Cycling conditions: 95 ℃ for 10 min, (95 ℃ for 15 s, 60 ℃ for 30 s, 72 ℃ for 1 min) x 15, 72 ℃ for 3 min. The 48 products each containing all 148 amplicons were analysed by the fragment analyser to ensure that the products fell within the expected range.

The 48 barcoded samples were pooled taking 1 µL from each sample, and purification of the sample pool was performed according to protocol. Briefly, Ampure XP beads were resuspended and 36 µL of the beads were added 12 µL of the pooled sample and 24 µL TE buffer. This solution was incubated at room temperature for 10 minutes and placed on a magnetic separator. The supernatant was discarded and beads were washed twice with 70% EtOH. The pellet was then air-dried for 10 minutes and resuspended in 40 µL DNA suspension buffer. The supernatant containing the cleaned fragments was transferred to a new tube.

The sample was analysed on the Agilent Bioanalyser to ensure that primers were successfully removed from the sample, and diluted to a final concentration of 2nM. The sample was then denatured by incubating with 0.2 N NaOH for 5 minutes at room temperature. The denatured sample was further diluted to 10pM in pre-chilled HT1 (Hybridization Buffer provided in the MiSeq reagent kit) and spiked with 1-2% Illumina PhiX control (Catalog # FC-110-3001) before loading on the MiSeq.

Table S5. Samples in real-time PCR validation.

acronym	type	Description
Ball1	Cell line	ALL (B cell)
CEM	Cell line	A (T cell)
DAUDI	Cell line	Burkitt's lymphoma (B cell)
EOL1	Cell line	Eosinophilic leukemia
HEL	Cell line	Erythroleukemia
HL60	Cell line	Promyelocytic leukemia
JURKAT	Cell line	T cell leukemia
KG1a	Cell line	AML
KU812	Cell line	CML (Basophilic cell line)
K562	Cell line	Erythroleukemia
L428	Cell line	Hodgkin's Lymphoma (myelomonocytic cell line?)
MEG01	Cell line	Megakaryoblastic leukemia
MOLT4	Cell line	ALL (T cell)
NALM1	Cell line	CML
NB4	Cell line	Promyelocytic leukemia
REH	Cell line	ALL (pro B cell)
RPMI_8226	Cell line	Plasmacytoma/Myeloma (B cell)
U937	Cell line	Histiocytic lymphoma (monoblastic cell line)
YT	Cell line	ALL (NK cell line)
NHB	Cell line	Bone cells?

Bone	Tissue	
Brain	Tissue	
Colon	Tissue	
Heart	Tissue	
Kidney	Tissue	
Liver	Tissue	
Lung	Tissue	
Muscle	Tissue	
Placenta	Tissue	
PBLs	Tissue	
Skin	Tissue	
Small intestine	Tissue	
Spleen	Tissue	
Stomach	Tissue	
Testis	Tissue	
Foetal Brain	Tissue	
Foetal Heart	Tissue	
Foetal Kidney	Tissue	
Foetal Liver	Tissue	
Foetal Lung	Tissue	
AML 95		acute myeloid leukemia (AML) samples
AML 96		acute myeloid leukemia (AML) samples
AML 97		acute myeloid leukemia (AML) samples
AML 98		acute myeloid leukemia (AML) samples
AML 99		acute myeloid leukemia (AML) samples
AML 100		acute myeloid leukemia (AML) samples
NTC		
Blank		negative control, no RNA used

5.3 Validation from sequencing results

Sequencing results were grouped by barcodes and qualified with Trimmomatic[16]. Reads were mapped back to the mature transcripts with Bowtie. A successful validation of one splice sites requires: 1) each base of the target regions (defined by the primer pairs) was covered by at least 5 reads and 2) the junction sites were covered by at least 5 reads bridging at least 10 bases at each side. Each splice site was checked in 48 tissues.

6 Exploring novel genes

We got 11,722 VHC transcripts and majority of them located in KNOWN coding regions. In other words, most of these transcripts were novel isoforms of KNOWN genes.

1,053 VMC transcripts from 673 loci (including 485 VHC transcripts from 351 loci) were found out of KNOWN gene regions. 782 VMC transcripts from 594 loci (including 312 VHC transcripts from 266 loci) remained after the pseudogenes were removed. For VHC transcripts, 173 transcripts were filtered out as transcribed pseudo-genes.

Almost all the remained transcripts overlapped with L1 repeat elements. 583 VMC transcripts from 442 loci (including 257 VHC transcripts from 224 loci) were fully covered by single L1 elements (Figure S5 A and B). In addition, 154 VMC transcripts from 128 loci (including 40 transcripts from 32 loci) overlapped partially with L1 elements (Figure S5 C and D).

Only 15 transcripts from 10 genes didn’t overlap with L1 elements. Details were shown in Figure S6.

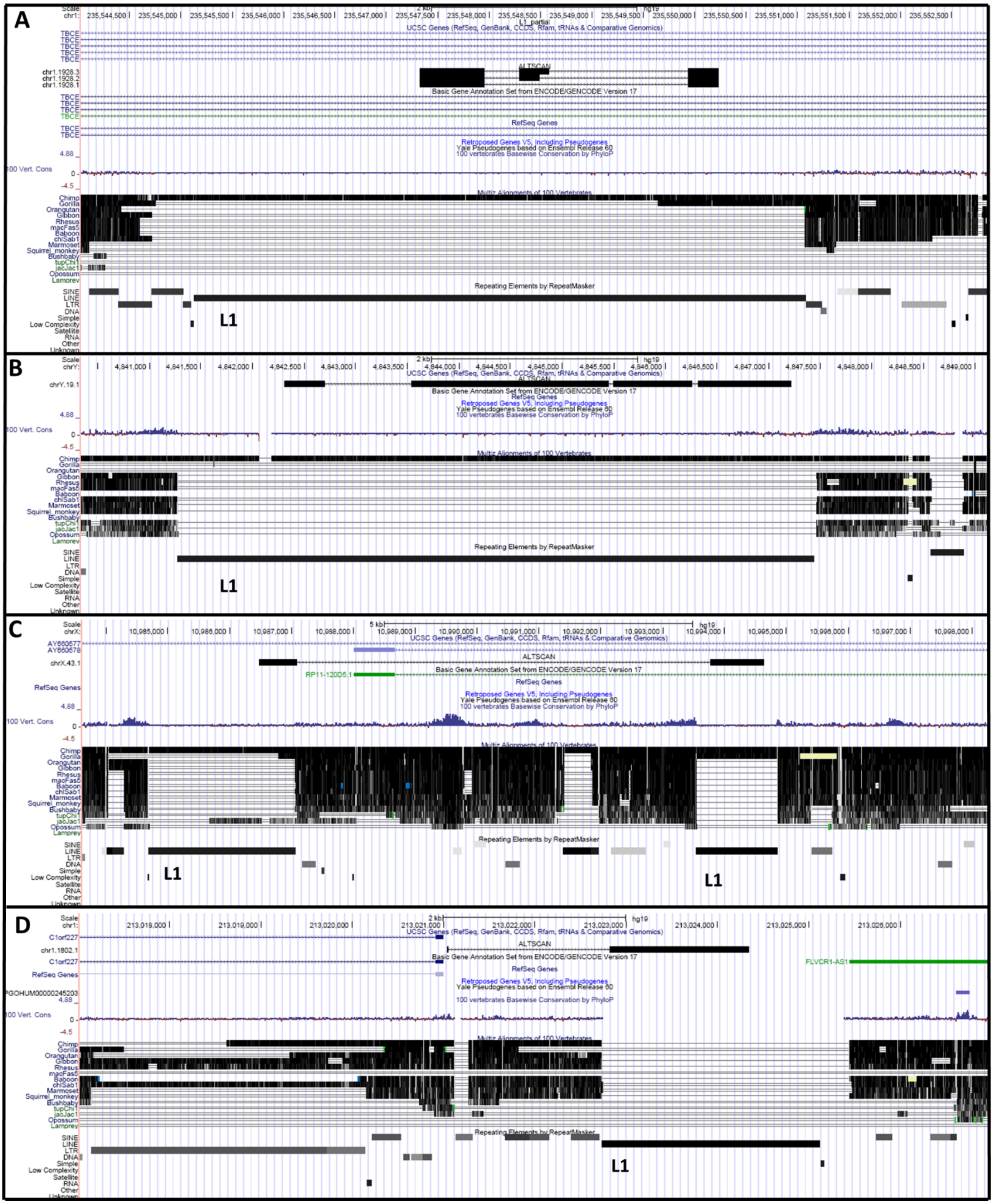

Figure S5. Illustrations of novel transcripts related with L1s. A and **B** showed transcripts fully covered by L1. **C** showed a transcript combined two different L1 elements. **D** was an example that L1 invaded the nearby genome. All these L1 elements were conserved only in chimpanzee and human or only found in human.

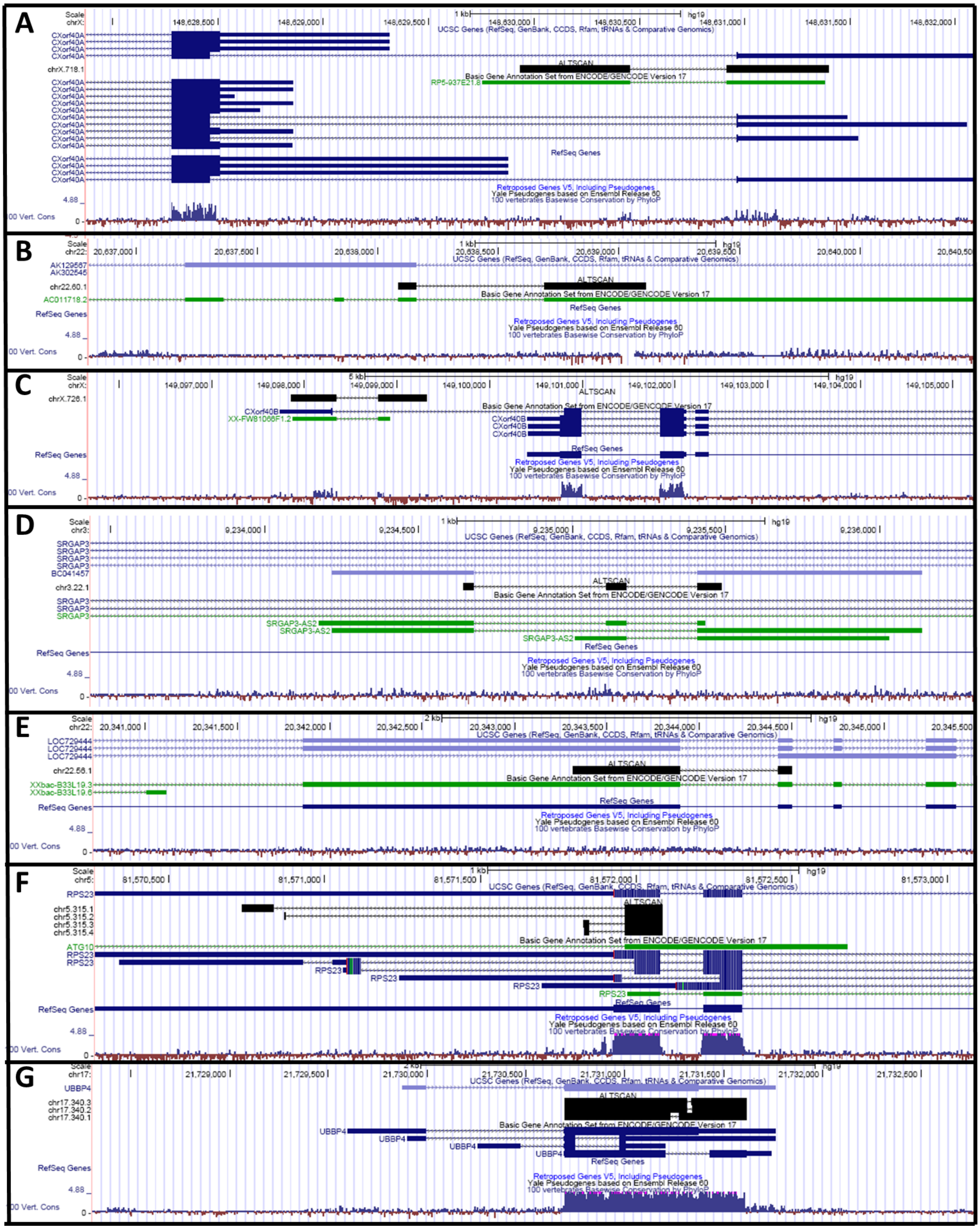
A
Scale
chrX:
148,628,500
148,629,000
148,629,500
148,630,000
148,630,500
148,631,000
148,631,500
148,632,000
1 kb
hg19
UCSC Genes (RefSeq, GenBank, CCDS, Rfam, tRNAs & Comparative Genomics)
CXorf40A
chrX.718.1
ALTSCAN
Basic Gene Annotation Set from ENCODE/GENCODE Version 17
RP5-937E21.8
RefSeq Genes
Retroposed Genes V5, Including Pseudogenes
Yale Pseudogenes based on Ensembl Release 60
100 vertebrates Basewise Conservation by PhyloP
4.88
100 Vert. Cons
0
B
chr22:
20,637,000
20,637,500
20,638,000
20,638,500
20,639,000
20,639,500
20,640,000
AK129567
AK302545
chr22.60.1
AC011718.2
C
chrX:
149,097,000
149,098,000
149,099,000
149,100,000
149,101,000
149,102,000
149,103,000
149,104,000
149,105,000
5 kb
chrX.726.1
CXorf40B
XX-FW81066F1.2
D
chr3:
9,234,000
9,234,500
9,235,000
9,235,500
9,236,000
SRGAP3
BC041457
chr3.22.1
SRGAP3-AS2
E
chr22:
20,341,000
20,341,500
20,342,000
20,342,500
20,343,000
20,343,500
20,344,000
20,344,500
20,345,000
20,345,500
2 kb
LOC729444
chr22.56.1
XXbac-B33L19.3
XXbac-B33L19.6
F
chr5:
81,570,500
81,571,000
81,571,500
81,572,000
81,572,500
81,573,000
RPS23
chr5.315.1
chr5.315.2
chr5.315.3
chr5.315.4
ATG10
G
chr17:
21,729,000
21,729,500
21,730,000
21,730,500
21,731,000
21,731,500
21,732,000
21,732,500
UBBP4
chr17.340.3
chr17.340.2
chr17.340.1

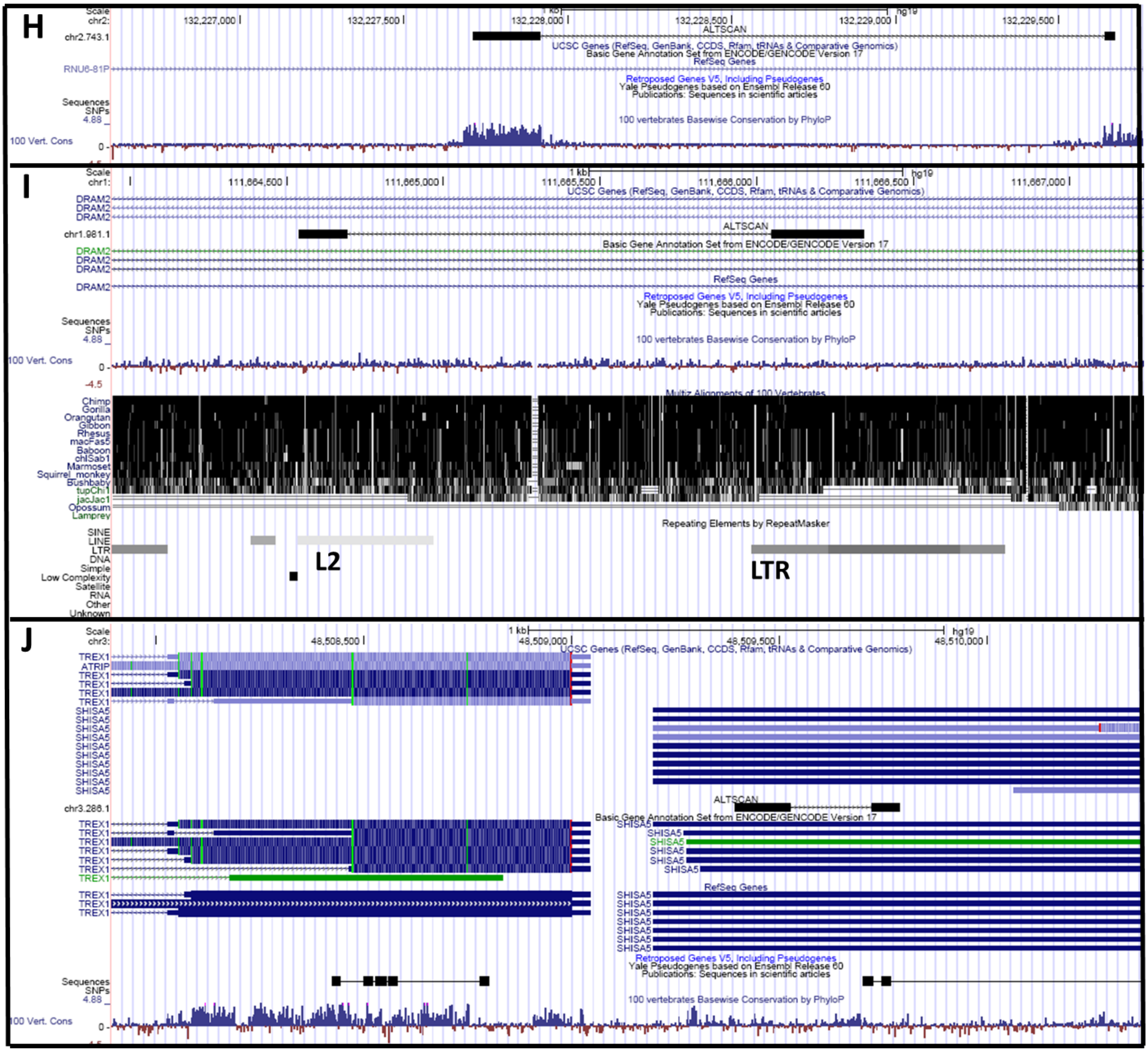

Figure S6. Novel genes overlapping no L1s. A-E showed 5 novel genes sharing the same splice junctions with previously annotated long non-coding RNAs. We found complete ORFs in these elements and showed their coding potentials. **F** showed a novel gene sharing a same splice site but different junctions with a previously annotated long non-coding RNAs. **G** showed a novel gene absent from GENCODE V12 but existing in GENCODE V17. Here we showed different splicing patterns. **H** showed a novel gene in the intron of gene RNU6-81P and its coding region was conserved. **I** showed a novel gene in the intron of DRAM2. This gene combined an L2 element and an LTR element. **J** showed a novel gene in the 3’ UTR region of SHISA5.

7 AS analysis

AS events are classified into seven categories: (1) exon skipping (cassette exons), an exon can be either included or excluded in the mature mRNA; (2) mutually exclusive exons, only one of the two or more adjacent exons can be included in the mature mRNA; (3) alternative splice acceptor sites; (4) alternative splice donor sites; (5) intron retention, where an intron can be either included or excluded in the mature mRNA; (6) alternative promoters (alternative first exons) and (7) alternative poly(A) (alternative last exons)[17-20]. The frequency of each AS event category varies. From an evolutionary perspective, intron retention is the most common category in lower metazoans and is common in fungi and protozoa; while the frequency of exon skipping increases in the eukaryotes[17,19]. In addition, exon skipping was considered to contribute most to the complexity of the proteome[17,19]. Alternative splice donor and acceptor sites are believed to be subfamilies of exon skipping and may represent an intermediate evolutionary stage.

We introduced 11,549 confident transcripts (after pseudo-transcripts were removed from the 11,722 VHC transcripts) and the majority came from known genes as we described above. Therefore we found more alternative transcripts for known genes. In addition, there are still 5,166 (29.9%) multi-exon genes with only one transcript in KNOWN dataset, and we found an alternative isoform for 488 of these 5,166 transcripts. We also checked the novel splice events (see Detections of AS events part for details) and found that cassette exons, alternative translation stop sites and alternative translation start sites contributed most (Table S6).

Table S6. Categorization of AS events in each dataset.

AS event types	KNOWN	KNOWN+VHC	KNOWN+VMC
Cassette exon	13,068 (18.23%)	17,590 (19.76%)	24,120(20.91%)
Alternative translation start site	20,042 (27.96%)	21,750 (24.44%)	25,396(22.01%)
Alternative translation stop site	19,080 (26.62%)	21,049 (23.65%)	23,803(20.63%)
Alternative donor site	8,273 (11.54%)	12,300 (13.82%)	18,234(15.80%)
Alternative acceptor site	8,281 (11.55%)	12,819 (14.40%)	19,048(16.51%)

Intron retention	2,538 (3.54%)	3,072 (3.45%)	4,378(3.80%)
Exclusive exon	392 (0.55%)	424 (0.48%)	392(0.34%)
Total	71,674 (100%)	89,004 (100%)	115,371(100%)

The numbers were measured by the number of involved splice sites. Thus, each event of cassette exon or intron retention involves 2 alternative splice sites and each event of exclusive exon involves 4 splice sites. Other events also involve a group of (at least 2) splice sites (see Supplemental materials for details).

7.1 Detection of AS events

Alternative splicing events are classified into seven categories: (1) exon skipping (cassette exons), where an exon can be either included or excluded in the mature mRNAs; (2) mutually exclusive exons, where only one of the two or more adjacent exons can be included in the mature mRNA; (3) alternative 5' splice sites; (4) alternative 3' splice sites; (5) intron retention, where an intron can be either included or excluded in the mature mRNAs; (6) alternative promoters (alternative first exons) and (7) alternative poly(A) sites (alternative last exons). Because we focus on coding regions, we change alternative promoters (alternative first exons) and alternative poly(A) sites (alternative last exons) to alternative translation start sites and translation stop sites. Here we show how we define and detect these events.

(1) Exon skipping (cassette exons)

A skipped exon (cassette exon) is defined as an exon that can exist or skip in the mature mRNAs. To detect this event, we compare every two transcript from a gene. Only when a target exon exists in one transcript and disappear in another, and both the upstream and downstream exons are exactly the same, we call it a cassette exon. As shown in Figure S7A, there are 5 transcripts in this gene. When comparing Transcript 1 with Transcript 2, the 2nd exon in Transcript 1 is captured. When Transcript 1 is compared with Transcript 5, the 2nd exon in Transcript 1 will not be captured, because the upstream exon is different in the acceptor site.

(2) Mutually exclusive exons

Exclusive exons are defined as a group of adjacent exons that only exact one can exist in one mature mRNA. First, we compare every two transcripts from a gene. As shown in Figure S7A, only the situation of Transcript 2 and 3 is captured. The 2nd exon from Transcript 2 and the 2nd exon from Transcript 3 are qualified as exclusive exon candidates. As in exon skipping detection, the upstream and downstream exons must be exactly the same. Then we'll go over all transcripts again, to see if these 2 exons satisfy the definition. For example, when checking Transcript 1, these 2 exons exist at the same time. Therefore, these candidates are abandoned.

(3) Alternative 5' or 3' splice sites (alternative donor or acceptor sites)

Two exons in each transcript must have a same splice site and a pair of different splice sites, then the different ones are defined as alternative donor or acceptor sites depending on their location. As shown in Figure S7A, when comparing Transcript 1 and Transcript 2, we capture a pair of acceptor sites, and when comparing Transcript 1 and Transcript 3, we capture a pair of donor sites. But when comparing Transcript 1 and Transcript 4, we capture nothing, because the downstream exons are not the same. In a similar way, when comparing Transcript 1 and Transcript 5, we capture nothing.

(4) Intron retention

Intron retention is defined as an intron that can appear in the mature mRNA. As shown in Figure S7B, when comparing Transcript 1 and Transcript 5, we capture one case at exon 2 of transcript 1. Of course, the donor site of upstream exon and acceptor site of downstream exon must be exactly the same as those of the exon in another transcript.

(5) Alternative translational start or stop site

It is just as its literal meaning, if two transcripts have different start or stop codons, these events will be reported.

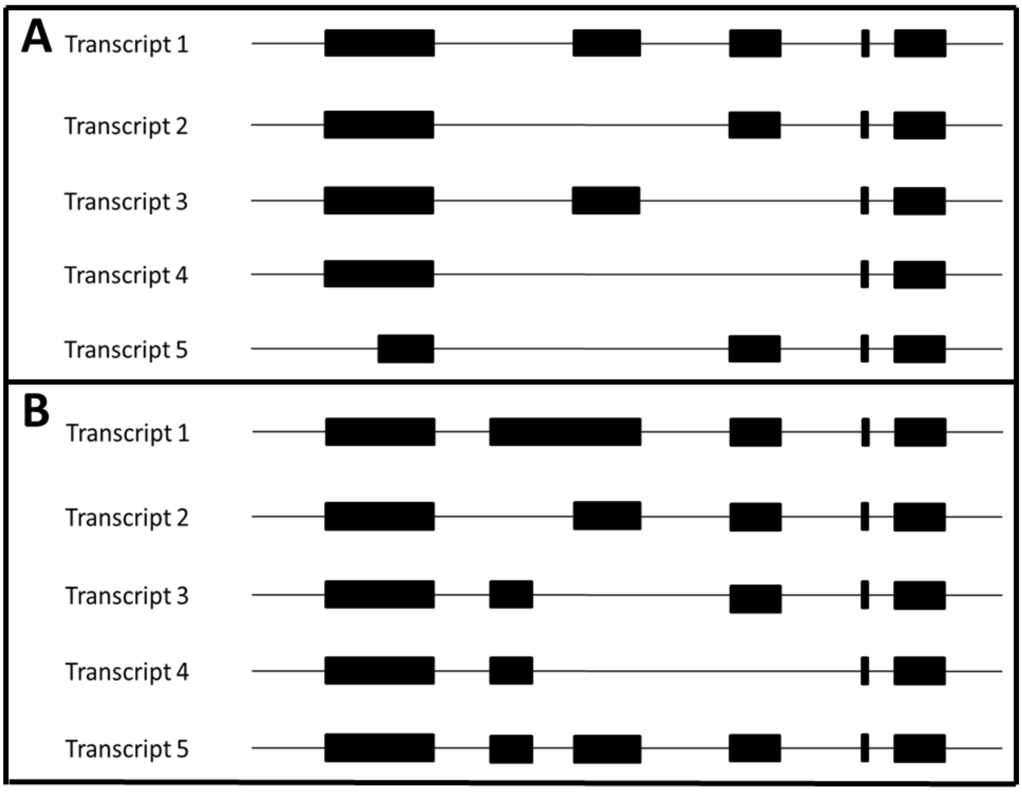

Figure S7. Illustrations in AS event detection. **A** and **B** showed two genes (groups of transcripts) to help explain how we detected AS events.

8 GO/KEGG analysis

8.1 GO analysis

A background function distribution is important for GO analysis. We checked the distribution of GO functions in KNOWN datasets and KNOWN+VHC or KNOWN+VMC datasets. Coding sequences of transcripts were first translated into protein sequences, then the protein sequences were aligned to the GO database using the NCBI blastp 2.26+. Only those alignments with E value ≤ 1e-5, identity≥30% and coverage of the query sequence ≥25% would be considered as matches. Sequences were first aligned to human proteins in GO database, and sequences failed to align to human proteins were then aligned to proteins of other species.

The comparison of distributions of GO functions were shown in Figure S8.

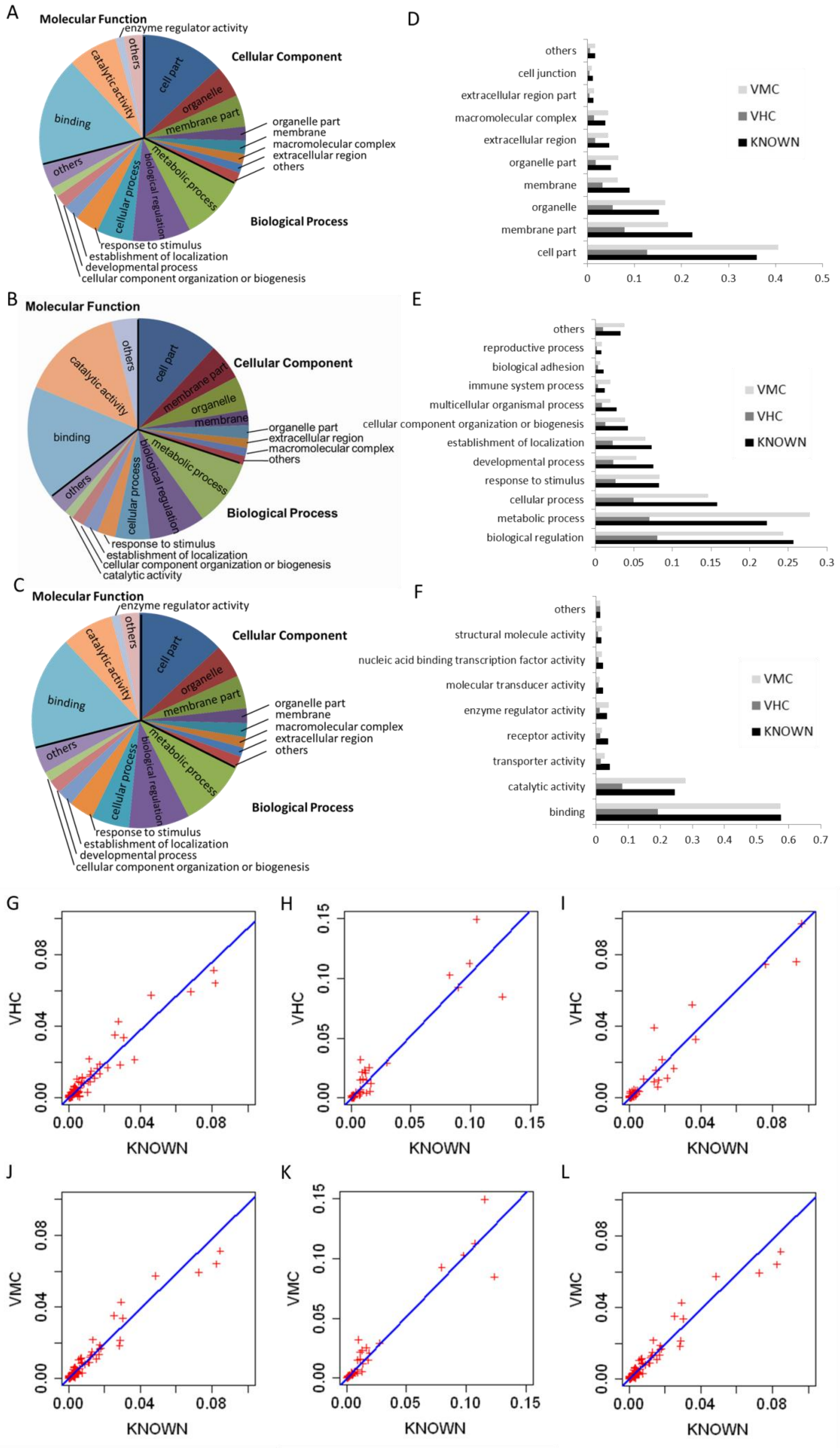
A
Molecular Function
enzyme regulator activity
others
catalytic activity
binding
others
Cellular Component
cell part
organelle
membrane part
organelle part
membrane
macromolecular complex
extracellular region
others
metabolic process
biological regulation
cellular process
Biological Process
response to stimulus
establishment of localization
developmental process
cellular component organization or biogenesis
B
Molecular Function
others
catalytic activity
binding
others
Cellular Component
cell part
membrane part
organelle
membrane
organelle part
extracellular region
macromolecular complex
others
metabolic process
biological regulation
cellular process
Biological Process
response to stimulus
establishment of localization
cellular component organization or biogenesis
catalytic activity
C
Molecular Function
enzyme regulator activity
others
catalytic activity
binding
others
Cellular Component
cell part
organelle
membrane part
organelle part
membrane
macromolecular complex
extracellular region
others
metabolic process
biological regulation
cellular process
Biological Process
response to stimulus
establishment of localization
developmental process
cellular component organization or biogenesis
D
others
cell junction
extracellular region part
macromolecular complex
extracellular region
organelle part
membrane
organelle
membrane part
cell part
VMC
VHC
KNOWN
0 0.1 0.2 0.3 0.4 0.5
E
others
reproductive process
biological adhesion
immune system process
multicellular organismal process
cellular component organization or biogenesis
establishment of localization
developmental process
response to stimulus
cellular process
metabolic process
biological regulation
VMC
VHC
KNOWN
0 0.05 0.1 0.15 0.2 0.25 0.3
F
others
structural molecule activity
nucleic acid binding transcription factor activity
molecular transducer activity
enzyme regulator activity
receptor activity
transporter activity
catalytic activity
binding
VMC
VHC
KNOWN
0 0.1 0.2 0.3 0.4 0.5 0.6 0.7
G
VHC
KNOWN
0.00 0.04 0.08
H
VHC
KNOWN
0.00 0.05 0.10 0.15
I
VHC
KNOWN
0.00 0.04 0.08
J
VMC
KNOWN
0.00 0.04 0.08
K
VMC
KNOWN
0.00 0.05 0.10 0.15
L
VMC
KNOWN
0.00 0.04 0.08

Figure S8. Distributions of KNOWN and VHC transcripts. A, distribution of GO first level functions for KNOWN transcripts; **B**, distribution of GO first level functions for KNOWN transcripts; **C**, distribution of GO first level functions for VMC transcripts. **D**, comparison of distributions of GO functions in Cellular component for VHC, VMC and KNOWN transcripts; **E**, comparison of distributions of GO functions in Biological process for VHC, VMC and KNOWN transcripts; **F**, comparison of distributions of GO functions in Molecular function for VHC, VMC and KNOWN transcripts. In **D**-**F**, y axis shows the functions and x axis shows the proportion of each function. In **A**-**F**, all functions with proportions < 0.01% were merged to "others". **G**, correlation of distribution of GO level 2 functions in Biological process between VHC and KNOWN transcripts; **H**, correlation of distribution of GO level 2 functions in Molecular function between VHC and KNOWN transcripts; **I**, correlation of distribution of GO level 2 functions in Cellular component between VHC and KNOWN transcripts. **J**, correlation of distribution of GO level 2 functions in Biological process between VMC and KNOWN transcripts; **K**, correlation of distribution of GO level 2 functions in Molecular function between VMC and KNOWN transcripts; **L**, correlation of distribution of GO level 2 functions in Cellular component between VMC and KNOWN transcripts. In **G-L**, x and y axis the proportion of functions. The Pearson correlations for **G-L** were 0.985, 0.950, 0.967, 0.989, 0.970 and 0.988, respectively.

8.2 Enrichment analysis

We checked if AS was enriched in some specific pathways or functions using KEGG [21] and GO annotation. KNOWN genes and ALTSCAN VMC genes were merged and then ranked by their transcript number. Enrichment analysis for the top quarter genes with the higheast number of transcripts (transcript number >5) were carried out with DAVID[22]. No significant enrichment were found in KEGG pathways. Enriched GO functions (adjusted p value with benjamini-hochberg method < 0.05) were shown in Table S7.

Table S7. Gene Ontology enrichment of genes with high number of transcripts.

	**GO functions**	
Molecular function	protein binding (6.0e-16)	transcription factor binding (5.60e-3)
		protein dimerization activity (2.60e-3)
		actin binding (1.70e-2)
		protein homodimerization activity (1.80e-2)
		cytoskeletal protein binding (2.00e-2)
	nucleoside binding (1.1e-3)	ATP binding (7.80e-4)

		adenyl ribonucleotide binding (1.40e-3)
		adenyl nucleotide binding (2.00e-3)
		purine nucleoside binding (3.30e-3)
	lipid binding	diacylglycerol binding (4.00e-2)
	GTPase regulator activity (3.0e-8)	small GTPase regulator activity (4.20e-8)
		Ras GTPase activator activity (6.20e-4)
	guanyl-nucleotide exchange factor activity (7.3e-5)	Rho guanyl-nucleotide exchange factor activity (2.90e-4)
		Ras guanyl-nucleotide exchange factor activity (3.30e-4)
	transcription cofactor activity (1.0e-2)	transcription activator activity (4.10e-2)
	protein kinase activity (3.7e-2)	protein serine/threonine kinase activity(4.20e-2)
Cellular component	neuron projection (4.7e-6)	synapse (4.80e-4)
		axon (1.10e-3)
		dendrite (8.80e-3)
		synaptosome (9.80e-3)
		postsynaptic density (2.60e-2)
	organelle (1.3e-4)	actin cytoskeleton (3.51e-8)
		clathrin-coated endocytic vesicle membrane (6.80e-3)
	membrane	coated pit (5.70e-3)
		extrinsic to membrane (6.80e-3)
		basolateral plasma membrane (1.70e-2)
		endomembrane system (4.60e-2)
		external side of plasma membrane (5.00e-2)
Biological process	membrane organization (5.9e-4)	membrane invagination (1.10e-2)
	biological adhesion (8.90e-3)	cell adhesion (9.40e-3)
	cell morphogenesis (9.8e-3)	cell morphogenesis involved in neuron differentiation (4.5e-2)
		cell projection morphogenesis (6.40e-3)
	establishment of localization (2.4e-2)	endocytosis (1.20e-2)
	organelle organization (4.6e-2)	cytoskeleton organization (1.50e-2)
	signal transduction	regulation of small GTPase mediated signal transduction (3.30e-3)
		regulation of Ras protein signal transduction (5.80e-3)
		regulation of Rho protein signal transduction (1.10e-2)

		transmembrane receptor protein tyrosine kinase signaling pathway (4.00e-2)
	transport	vesicle-mediated transport (1.90e-2)
	protein modification process	protein phosphorylation (1.40e-2)

The adjusted p-values with benjamini-hochberg method are shown in brackets.

9 Detection of novel proteins

9.1 Annotated dataset construction

A Target/decoy database was used for Mass Spectrum querying. The Target database contains the Refseq protein sequences, the predicted Refseq protein sequences and the protein sequences translated from ALTSCAN VMC transcripts. The decoy dataset was built by reversing the target database.

9.2 Database search

The 900 mass spectrum files from breast cancer were downloaded from CPTAC website (https://cptac-data-portal.georgetown.edu/cptacPublic/) and were used to query the Target/decoy database. The X!Tandem search engine was used to analyze the MS files. The searching parameters were set as default except fragment/parent monoisotopic mass errors were set to 20 ppm and maximum missed cleavage sites were set to 1. The 900 XML output files were then parsed using python scripts. For each output file, the results were sorted by hypescores from high to low and sorted by e-value from low to high. To reduce the false positively identified peptides, we set the FDR value to 10^{-6}. Here, the FDR were defined by FDR=2*F/(T+F), where F is the number of spectrums which were scored to the decoy proteins and T represents the number of rest spectrums.

9.3 Novel peptides and novel proteins identification

The peptides after FDR controlling were used for further analysis. Peptides that can score to the ALTSCAN VMC sequences but cannot scored to Refseq sequences were identified as the preliminary novel peptides. These preliminary peptides were further identified using blastp method to get the final novel peptides, to make sure they are not of 100% identical to the Refseq protein sequences. A protein that can be mapped by at least two identified peptide including at least one novel peptide was defined to a novel protein. Novel proteins and their supported novel peptides are shown in Table S8.

Table S8. Novel proteins detected from shotgun proteomics datasets.

Novel protein(Altscan id)	Novel peptide	Gene symbol
chr1.600.2	DGYELSPTAAANFTR(24)	PSMB2
chr1.1131.1/chr12.527.1/ chr13.516.1/chrX.229.1 et al.	CDQLEER(2)	L1 elements
chr11.1096.158	SGSFSELYTLSLQEPSRR(2)<br>SGSFSELYTLSLQEPSR(1)	SIPA1
chr11.1490.3	PECMLSNTTAIAEAWAR(3)<br>NHLISQIVSSITASLR(1)	Unknown
chr11.516.1	DFIMDNCEELIPEYLNFIR(3)	Unknown
chr12.304.6	PVCVECFSDYPPLGR(4)<br>MSTKIGGIGTVPVGR(1)	Unknown
chr16.35.29	YMADMDELFSQKR(5)	STUB1
chr16.449.151/chr16.449.27/ chr16.449.66/chr16.449.87	ELMVGAGPTQTVVMHR(1)	EIF3C
chr18.385.20	EGTAEMSSILEER(8)	ATP5A1
chr18.385.31	PVIETQAAPMAIEEQVAVIYAGVR(13)	ATP5A1
chr19.1402.10	ESEKPAQSLGEEAEPADR(2)	PPP1R12C
chr19.238.1	AAGFAVTGCTCGSACGSWDVR(8)	RETN
chr19.262.101	INGGGGGSVPGIER(59)	HNRNPM
chr19.443.4	CQALCGCTVNVPTLDGR(3)	DNAJB1
chr19.444.10	MDLNLSLLAR(2)	TECR
chr19.799.4	LSHPNLPR(1)	HSPB6
chr19.930.11	FCGTPEYLAPEVSGVLEDNDYGR(5)	AKT2
chr2.103.15	AWYCDDVIELTPSNFNR(23)<br>TAWYCDDVIELTPSNFNR(1)	PDIA6
chr2.2331.4	LVRSDQCTGLQGFLVFHSFGGGTGSGFTSLLMER(2)	TUBA4A
chr2.246.20	DLTGSPGKDGQEGPIGPPGVEGAPAAPVPQVAFSAALSLPR(15)	EMILIN1
chr2.779.9	GSTVGIVLDSGDGVTHNVPIYEGYALPHAIMR(36)	ACTG2

chr3.2186.20	SLYIIIGR(2)	EIF4A2
chr3.571.2/chr3.571.3/chr3.571.4/ chr3.571.5	LHPSGPIEQLPDYNR(1)	UQCRC1
chr4.1202.3/ chr4.1202.4	GDAAIVDMVPGKPVYVESFSDYPPLGR(3)	Unknown
chr5.1245.2/ chr5.1245.5	CKLTTLGNLTPSSTVFFCCDMQER(2)	ISOC1
chr5.1361.17/ chr5.1361.19	CELSSSVQVQQTVQDLFGR(1)	HSPA9
chr5.800.1/ chr5.800.2	EIKGIQLGR(1)	L1 elements
chr5.921.5	TASLGLR(1)	Unknown
chr6.1507.4/chr6.1507.6/ chr6.1507.8	KSINVIQNINR(1)	L1 elements
chr6.263.2	MVNPTMFFDIAVDGEPLGR(17)	Unknown
chr7.109.7	PMYQEESNLSLQALESR(1) MYQEESNLSLQALESR(5)	AIMP2
chr8.1540.9	FPLHGRGTR(1)	PYCRL
chrX.1417.9	GPPRDDGGYSMNFNMSSSR(1)	FLNA
chrX.1600.33	VHSPSGALEECYVTEIDQGKDS(1)	G6PD
chrX.1613.5	YKVPYR(1)	ELMO2
chr20.565.13	HLNPLDLPVTHVIR(2)	KRT6B

The numbers in the brackets following the peptides show the detected times.

11 Accessing software and data

ALTSCAN is available and can be downloaded from

http://cbb.sjtu.edu.cn/~ccwei/pub/software/ALTSCAN/ALTSCAN.php.

VHC and VMC datasets can be downloaded from

http://cbb.sjtu.edu.cn/~ccwei/pub/software/ALTSCAN/Rna-seq.htm.

12 References

1. Burge, C. & Karlin, S. Prediction of complete gene structures in human genomic DNA. *J Mol Biol* **268**, 78-94 (1997).
2. Keibler, E. & Brent, M.R. Eval: A software package for analysis of genome annotations. *BMC Bioinformatics* **4**, 50 (2003).
3. Pruitt, K.D., Tatusova, T. & Maglott, D.R. NCBI reference sequences (RefSeq): a curated non-redundant sequence database of genomes, transcripts and proteins. *Nucleic Acids Res* **35**, D61-5 (2007).
4. Benson, D.A., Karsch-Mizrachi, I., Lipman, D.J., Ostell, J. & Wheeler, D.L. GenBank: update. *Nucleic Acids Res* **32**, D23-6 (2004).

5. Gross, S.S. & Brent, M.R. Using multiple alignments to improve gene prediction. *J Comput Biol* **13**, 379-93 (2006).
6. Blanco, E., Parra, G. & Guigo, R. Using geneid to identify genes. *Curr Protoc Bioinformatics* **Chapter 4**, Unit 4 3 (2007).
7. Stanke, M. et al. AUGUSTUS: ab initio prediction of alternative transcripts. *Nucleic Acids Res* **34**, W435-9 (2006).
8. Stanke, M., Schoffmann, O., Morgenstern, B. & Waack, S. Gene prediction in eukaryotes with a generalized hidden Markov model that uses hints from external sources. *BMC Bioinformatics* **7**, 62 (2006).
9. Slater, G.S. & Birney, E. Automated generation of heuristics for biological sequence comparison. *BMC Bioinformatics* **6**, 31 (2005).
10. Schweikert, G. et al. mGene: accurate SVM-based gene finding with an application to nematode genomes. *Genome Res* **19**, 2133-43 (2009).
11. Sperisen, P. et al. trome, trEST and trGEN: databases of predicted protein sequences. *Nucleic Acids Res* **32**, D509-11 (2004).
12. Steijger, T. et al. Assessment of transcript reconstruction methods for RNA-seq. *Nat Methods* **10**, 1177-84 (2013).
13. Engstrom, P.G. et al. Systematic evaluation of spliced alignment programs for RNA-seq data. *Nat Methods* **10**, 1185-91 (2013).
14. Dai, M. et al. NGSQC: cross-platform quality analysis pipeline for deep sequencing data. *BMC Genomics* **11 Suppl 4**, S7 (2010).
15. Langmead, B., Trapnell, C., Pop, M. & Salzberg, S.L. Ultrafast and memory-efficient alignment of short DNA sequences to the human genome. *Genome Biol* **10**, R25 (2009).
16. Alquezar-Planas, D.E. et al. Discovery of a divergent HPIV4 from respiratory secretions using second and third generation metagenomic sequencing.
17. Keren, H., Lev-Maor, G. & Ast, G. Alternative splicing and evolution: diversification, exon definition and function. *Nat Rev Genet* **11**, 345-55 (2010).
18. Irimia, M. & Blencowe, B.J. Alternative splicing: decoding an expansive regulatory layer. *Curr Opin Cell Biol* **24**, 323-32 (2012).
19. Kim, E., Goren, A. & Ast, G. Alternative splicing: current perspectives. *Bioessays* **30**, 38-47 (2008).
20. Rogozin, I.B., Wolf, Y.I., Sorokin, A.V., Mirkin, B.G. & Koonin, E.V. Remarkable interkingdom conservation of intron positions and massive, lineage-specific intron loss and gain in eukaryotic evolution. *Curr Biol* **13**, 1512-7 (2003).
21. Kanehisa, M., Goto, S., Sato, Y., Furumichi, M. & Tanabe, M. KEGG for integration and interpretation of large-scale molecular data sets. *Nucleic Acids Res* **40**, D109-14 (2012).
22. Huang da, W., Sherman, B.T. & Lempicki, R.A. Systematic and integrative analysis of large gene lists using DAVID bioinformatics resources. *Nat Protoc* **4**, 44-57 (2009).
